# The burden of chronic respiratory diseases and their heterogeneity across the states of India: the Global Burden of Disease Study 1990–2016

**DOI:** 10.1016/S2214-109X(18)30409-1

**Published:** 2018-09-12

**Authors:** India State-Level Disease Burden Initiative CRD Collaborators, Sundeep Salvi, G Anil Kumar, R S Dhaliwal, Katherine Paulson, Anurag Agrawal, Parvaiz A Koul, P A Mahesh, Sanjeev Nair, Virendra Singh, Ashutosh N Aggarwal, D J Christopher, Randeep Guleria, B V Murali Mohan, Surya K Tripathi, Aloke G Ghoshal, R Vijai Kumar, Ravi Mehrotra, D K Shukla, Eliza Dutta, Melissa Furtado, Deeksha Bhardwaj, Mari Smith, Rizwan S Abdulkader, Monika Arora, Kalpana Balakrishnan, Joy K Chakma, Pankaj Chaturvedi, Sagnik Dey, Deesha Ghorpade, Scott Glenn, Prakash C Gupta, Tarun Gupta, Sarah C Johnson, Tushar K Joshi, Michael Kutz, Manu R Mathur, Prashant Mathur, Pallavi Muraleedharan, Christopher M Odell, Sanghamitra Pati, Yogesh Sabde, Dhirendra N Sinha, K R Thankappan, Chris M Varghese, Geetika Yadav, Stephen S Lim, Mohsen Naghavi, Rakhi Dandona, K Srinath Reddy, Theo Vos, Christopher J L Murray, Soumya Swaminathan, Lalit Dandona

## Abstract

**Background:**

India has 18% of the global population and an increasing burden of chronic respiratory diseases. However, a systematic understanding of the distribution of chronic respiratory diseases and their trends over time is not readily available for all of the states of India. Our aim was to report the trends in the burden of chronic respiratory diseases and the heterogeneity in their distribution in all states of India between 1990 and 2016.

**Methods:**

Using all accessible data from multiple sources, we estimated the prevalence of major chronic respiratory diseases and the deaths and disability-adjusted life-years (DALYs) caused by them for every state of India from 1990 to 2016 as part of the Global Burden of Diseases, Injuries, and Risk Factors Study (GBD) 2016. We assessed heterogeneity in the burden of chronic obstructive pulmonary disease (COPD) and asthma across the states of India. The states were categorised into four groups based on their epidemiological transition level (ETL). ETL was defined as the ratio of DALYs from communicable diseases to those from non-communicable diseases and injuries combined, with a low ratio denoting high ETL and vice versa. We also assessed the contribution of risk factors to DALYs due to COPD. We compared the burden of chronic respiratory diseases in India against the global average in GBD 2016. We calculated 95% uncertainty intervals (UIs) for the point estimates.

**Findings:**

The contribution of chronic respiratory diseases to the total DALYs in India increased from 4·5% (95% UI 4·0–4·9) in 1990 to 6·4% (5·8–7·0) in 2016. Of the total global DALYs due to chronic respiratory diseases in 2016, 32·0% occurred in India. COPD and asthma were responsible for 75·6% and 20·0% of the chronic respiratory disease DALYs, respectively, in India in 2016. The number of cases of COPD in India increased from 28·1 million (27·0–29·2) in 1990 to 55·3 million (53·1–57·6) in 2016, an increase in prevalence from 3·3% (3·1–3·4) to 4·2% (4·0–4·4). The age-standardised COPD prevalence and DALY rates in 2016 were highest in the less developed low ETL state group. There were 37·9 million (35·7–40·2) cases of asthma in India in 2016, with similar prevalence in the four ETL state groups, but the highest DALY rate was in the low ETL state group. The highest DALY rates for both COPD and asthma in 2016 were in the low ETL states of Rajasthan and Uttar Pradesh. The DALYs per case of COPD and asthma were 1·7 and 2·4 times higher in India than the global average in 2016, respectively; most states had higher rates compared with other locations worldwide at similar levels of Socio-demographic Index. Of the DALYs due to COPD in India in 2016, 53·7% (43·1–65·0) were attributable to air pollution, 25·4% (19·5–31·7) to tobacco use, and 16·5% (14·1–19·2) to occupational risks, making these the leading risk factors for COPD.

**Interpretation:**

India has a disproportionately high burden of chronic respiratory diseases. The increasing contribution of these diseases to the overall disease burden across India and the high rate of health loss from them, especially in the less developed low ETL states, highlights the need for focused policy interventions to address this significant cause of disease burden in India.

**Funding:**

Bill & Melinda Gates Foundation; and Indian Council of Medical Research, Department of Health Research, Ministry of Health and Family Welfare, Government of India.

## Introduction

Chronic respiratory diseases include chronic obstructive pulmonary disease (COPD), asthma, pneumoconiosis, interstitial lung diseases, and pulmonary sarcoidosis. Of these diseases, COPD and asthma are the most common.^1^ COPD is one of the leading non-communicable causes of death globally, as well as in India.^1^, ^2^, ^3^ India has a population of 1·3 billion people living in 29 states and seven union territories, many of which have populations as large as some countries, and which often vary widely in terms of ecology, economy, and demography, all of which affect respiratory health.^2^, ^4^ The Sustainable Development Goals aim to reduce premature mortality from non-communicable diseases by a third by 2030 through prevention and treatment.^5^ The National Health Policy of India 2017 recommends that premature mortality from non-communicable diseases, including chronic respiratory diseases, should be reduced by 25% by 2025.^6^

**Research in context**

**Evidence before this study**

We searched PubMed and publicly available reports for estimates of the burden of chronic respiratory diseases, including COPD and asthma, across the states of India using the search terms “asthma”, “burden”, “cause of death”, “chronic respiratory disease”, “death”, “DALY”, “epidemiology”, “India”, “lung diseases”, “morbidity”, “mortality”, “prevalence”, “pulmonary disease, chronic obstructive”, and “trends” on March 26, 2018, without language or other publication restrictions. Although previous attempts have been made to assess the burden of COPD and asthma in some parts of the country, there have been no reports summarising the burden of chronic respiratory diseases or a comprehensive compilation of the prevalence, deaths, and DALYs for COPD and asthma across all states of India over a prolonged period of time.

**Added value of this study**

This report highlights the disproportionate burden of chronic respiratory diseases in India, which has 18% of the world's population, but 32% of the global DALYs from these diseases. We provide a comprehensive assessment of the burden of COPD and asthma in every state of India from 1990 to 2016, based on all accessible data and using the standardised Global Burden of Diseases, Injuries, and Risk Factors Study methodology. The highest rates of age-standardised DALYs from COPD and asthma in 2016 were in the relatively less developed states in north India. An important finding of this study is that most states in India have higher DALYs rates due to COPD and asthma than do other locations around the world at similar levels of sociodemographic development. The finding that DALYs per person with COPD and asthma in India are 1·7 and 2·4 times higher than the global average, respectively, points to the need to improve the detection and management of these diseases. Furthermore, the findings highlight that air pollution was the leading risk factor for COPD in India in 2016, being responsible for more COPD burden than smoking.

**Implications of all the available evidence**

This comprehensive assessment of the burden of chronic respiratory diseases in every state of India from 1990 to 2016 points to the clear need for health system strategies to address these diseases, given that there is currently no large-scale systematic effort to control this burden at the population level in India. While nationwide strategies are needed to bring more prominence to the control of COPD and asthma, the state-level findings can provide useful guidance on how to modify the efforts based on the disease burden and risk factor trends in each state of India.

Health is a state subject in the Indian federal structure, with most health expenditure coming from state budgets.^7^ Accurate and comprehensive data on what is driving chronic respiratory disease burden in each state of India is therefore crucial for policy makers to be able to make the best informed decisions possible for improving respiratory health. Some attempts have been made previously to compile the burden of COPD and asthma using studies from different parts of India.^8^, ^9^, ^10^, ^11^ However, a systematic understanding of the distribution of chronic respiratory diseases and their trends over time is not readily available for all of the states of India.

In 2017, the India State-Level Disease Burden Initiative reported a varied epidemiological transition occurring among the states of India from 1990 to 2016 as part of the Global Burden of Diseases, Injuries, and Risk Factors Study (GBD) 2016.^2^, ^3^ Here, we report changes in the burden of chronic respiratory diseases, in particular the heterogeneity in the distribution of COPD and asthma, across all states of India between 1990 and 2016.

## Methods

### Overview

The India State-Level Disease Burden Initiative has reported the overall trends of diseases, injuries, and risk factors for every state of India from 1990 to 2016.^2^, ^3^ This analysis was done as part of GBD 2016, which used all accessible data to estimate disease burden due to 333 diseases and injuries and 84 risk factors. The India State-Level Disease Burden Initiative was supported by the efforts of several expert groups and a large network of collaborators who identified and accessed all available data sources, assessed their scope and quality for inclusion, and participated in the analysis and interpretation of the findings. The Health Ministry Screening Committee at the Indian Council of Medical Research and the ethics committee of the Public Health Foundation of India approved the work of this initiative. Detailed descriptions of the metrics and analytical approaches used in GBD 2016 have been reported elsewhere.^1^, ^12^, ^13^, ^14^, ^15^, ^16^ Here, we report findings on the burden of chronic respiratory diseases, in particular COPD and asthma, and their heterogeneity across the states of India from 1990 to 2016. The GBD 2016 methods relevant to this Article are described in the appendix (pp 3–22), and a summary of the key points follows.

### Estimation of prevalence and years lived with disability

The prevalence of chronic respiratory diseases was estimated by location, age, sex, and year using DisMod-MR version 2.1, an updated Bayesian regression analytical tool used in GBD to estimate non-fatal health outcomes.^14^ The major data inputs for estimating the prevalence of chronic respiratory diseases in India were population-representative surveys and cohort studies, including published and unpublished studies (appendix pp 23–33). The Burden of Obstructive Lung Disease Initiative Survey (BOLD) was one of the major data sources used to estimate COPD prevalence in India. The Indian Study on Epidemiology of Asthma, Respiratory Symptoms and Chronic Bronchitis in adults (INSEARCH), and the International Study of Asthma and Allergy in Childhood (ISAAC) were the major data sources used to estimate asthma prevalence in India.^17^, ^18^, ^19^

All available and accessible data on prevalence, incidence, and remission were used in the modelling if they met the quality and inclusion criteria. The estimation included ascertainment of the severity distribution of sequelae, incorporation of disability weights, and comorbidity adjustment of sequelae. Years lived with disability (YLDs) were calculated by multiplying the prevalence of each sequela by its disability weight for the corresponding health state.

GBD uses covariates, which are explanatory variables that have a known association with the outcome of interest, to arrive at the best possible estimate when data on the outcome are scarce but data on the covariates are available.^12^, ^13^, ^14^, ^15^, ^16^ This approach was part of the estimation process for the findings presented in this report.

### Estimation of deaths, years of life lost, and disability-adjusted life-years

Deaths due to chronic respiratory diseases were estimated with Cause of Death Ensemble modelling. For this estimation, the completeness of death records was assessed using statistical models, and recorded deaths were mapped with the International Classification of Diseases versions 9 and 10 codes to enable a consistent classification of the causes of death. The garbage codes for specific chronic respiratory disease causes of death were redistributed proportionally using regression models. The CoDCorrect algorithm was used to adjust the estimates such that the predicted number of deaths from the components (eg, COPD deaths) added up to the total for chronic respiratory diseases, and the sum of deaths from all causes added up to the total deaths.^1^, ^12^ Years of life lost (YLLs) due to premature death were estimated using the GBD normative standard life expectancy at each age. Disability-adjusted life-years (DALYs) were calculated as the sum of YLLs and YLDs for each cause.

The major data inputs for the estimates of mortality due to chronic respiratory diseases included Sample Registration System cause of death data, Medical Certification of Cause of Death data, and other verbal autopsy studies (appendix pp 23–33).

### Estimation of risk factor exposure and attributable disease burden

The GBD comparative risk assessment framework was used to estimate risk factor exposure related to chronic respiratory diseases and the attributable disease burden.^16^ Exposure data for risk factors with a categorical or continuous distribution were collated from all available data sources that could be accessed, including survey and other data, adjusted by use of age–sex splitting, and strengthened with the incorporation of covariates for modelling. The modelling approach integrated multiple data inputs and borrowed information across age, time, and location to produce the best possible estimates of risk exposure by location, age, sex, and year. For each risk factor, the theoretical minimum risk exposure level was established as the lowest level of risk exposure below which its relationship with a disease outcome is not supported by the available evidence.^16^ Estimates of mean risk factor exposure, strengthened by covariates, were used to calculate summary exposure values for each risk, a metric ranging from 0% to 100% to describe the risk-weighted exposure for a population or risk-weighted prevalence of exposure.

Estimation of attributable disease burden included ascertainment of relative risk of disease outcomes for risk exposure–disease outcome pairs with sufficient evidence of a causal relationship in randomised controlled trials, prospective cohorts, or case-control studies, as assessed with an approach similar to the World Cancer Research Fund grading system, and then estimation of population attributable fractions for diseases caused by each risk factor.^16^ Estimates of deaths and DALYs attributable to each risk factor were produced by location, age, sex, and year. A detailed description of exposure and attributable disease burden estimation for the major risk factors associated with chronic respiratory diseases, including GBD exposure definitions and statistical modelling, is described in the appendix (pp 3–22), and provided elsewhere.^16^

The major data inputs for risk factors for chronic respiratory diseases in India included nationwide household health surveys, air pollution monitoring and satellite data, youth and adult tobacco surveys, occupational surveys of the National Sample Survey Organisation, and several other large population-level surveys (appendix pp 23–33).

### Analysis presented in this paper

Findings are reported for 31 geographical units in India: 29 states, the Union Territory of Delhi, and the union territories other than Delhi (combining the six smaller union territories of Andaman and Nicobar Islands, Chandigarh, Dadra and Nagar Haveli, Daman and Diu, Lakshadweep, and Puducherry). The states of Chhattisgarh, Uttarakhand, and Jharkhand were created from existing larger states in 2000, and the state of Telangana was created in 2014. For trends from 1990 onward, the data for these four new states were disaggregated from their parent states on the basis of data from the districts that now constitute these states. The findings are also presented for four groups of states based on their epidemiological transition level (ETL), as described previously.^2^ Briefly, ETL state groups were defined on the basis of the ratio of DALYs from communicable, maternal, neonatal, and nutritional diseases to those from non-communicable diseases and injuries combined in 2016, with a lower ratio indicating higher ETL: low ETL (ratio 0·56–0·75), lower-middle ETL (0·41–0·55), higher-middle ETL (0·31–0·40), and high ETL (<0·31).^2^ We have previously reported that epidemiological transition ratios of the states of India have a significant inverse relation with the Socio-demographic Index (SDI) calculated by GBD based on income, education, and fertility levels, suggesting broad correspondence between the ETL state groups and sociodemographic development levels.^2^

We present findings on deaths and DALYs due to chronic respiratory diseases and each of the component causes by sex in India in 2016. We present comparisons of the prevalence of, and deaths and DALYs due to COPD and asthma in the states of India from 1990 to 2016. We report age-specific and sex-specific prevalence of COPD and asthma in India in 2016. We report the change in case-fatality rates for COPD and asthma in the ETL state groups from 1990 to 2016. We present the COPD DALYs attributable to risk factors in India and at the level of the ETL state groups in 2016. Additionally, we assessed the ratio of the observed-to-expected DALY rates of COPD and asthma in each state as compared with other locations worldwide at a similar level of SDI. We also compared the prevalence and DALYs per case of COPD and asthma in India versus the global average in GBD 2016.^20^

We present both crude and age-standardised estimates as relevant. Crude estimates provide the actual situation in each state, which is useful for policy makers, and age-standardised estimates allow comparisons over time and between states after adjusting for the differences in the age structure of the population. Age-standardised rates were based on the GBD global reference population.^12^ Estimates are reported with 95% uncertainty intervals (UIs) where relevant. These were based on 1000 runs of the models for each quantity of interest, with the mean considered as the point estimate and the 2·5th and 97·5th percentiles considered as the 95% UI (appendix p 22).^12^, ^13^, ^14^, ^15^, ^16^

### Role of the funding source

Some staff of the Indian Council of Medical Research are coauthors on this paper, having contributed to various aspects of the study and analysis. The other funder of the study had no role in the study design, data collection, data analysis, data interpretation, or writing of this paper. The corresponding author had full access to all the data in the study, and had final responsibility for the decision to submit for publication.

## Results

Chronic respiratory diseases were responsible for 10·9% (95% UI 10·0–12·0) of the total deaths and 6·4% (5·8–7·0) of the total DALYs in India in 2016 (table 1), as compared with 9·6% (8·2–10·5) and 4·5% (UI 4·0–4·9), respectively, in 1990.^21^ Of the total global DALYs due to chronic respiratory diseases in 2016, 32% were in India.^20^ COPD and asthma were the predominant chronic respiratory diseases, with COPD contributing 75·6% of the total DALYs due to chronic respiratory diseases in 2016 and asthma 20·0%. After ischaemic heart disease, COPD was the second leading cause of disease burden in India,^2^ contributing 8·7% (7·8–9·5) of the total deaths and 4·8% (4·3–5·3) of the total DALYs. The proportions of deaths and DALYs from pneumoconiosis were significantly higher in men than in women, but the proportions for COPD, asthma, and interstitial lung disease and pulmonary sarcoidosis were similar among men and women (table 1).

**Table 1:** Percentage of deaths and DALYs due to each cause under the category of chronic respiratory diseases in India, 2016

		**Percentage of total deaths (95% UI)**			**Percentage of total DALYs (95% UI)**		
		Both sexes	Male	Female	Both sexes	Male	Female
Chronic respiratory diseases		10·9% (10·0–12·0)	10·8% (10·0–11·4)	11·1% (9·4–13·5)	6·4% (5·8–7·0)	6·7% (6·2–7·1)	6·0% (5·3–7·1)
	COPD	8·7% (7·8–9·5)	8·7% (7·6–9·7)	8·6% (7·1–10·5)	4·8% (4·3–5·3)	5·2% (4·6–5·7)	4·4 % (3·8–5·3)
	Asthma	1·9% (1·2–2·5)	1·6% (0·9–2·6)	2·1% (1·4–3·2)	1·3% (0·9–1·6)	1·2% (0·8–1·6)	1·4% (1·0–1·9)
	Interstitial lung disease and pulmonary sarcoidosis	0·28% (0·16–0·40)	0·27% (0·14–0·42)	0·28% (0·15–0·47)	0·14% (0·08–0·20)	0·14% (0·08–0·22)	0·13% (0·07–0·22)
	Pneumoconiosis	0·04% (0·03–0·05)	0·05% (0·04–0·07)	0·01% (0·01–0·02)	0·02% (0·01–0·02)	0·03% (0·02–0·04)	0·01% (0·00–0·01)
	Other chronic respiratory diseases	0·09% (0·05–0·11)	0·12% (0·06–0·17)	0·04% (0·02–0·07)	0·13% (0·11–0·16)	0·16% (0·12–0·20)	0·10% (0·08–0·12)

DALY=disability-adjusted life-year. COPD=chronic obstructive pulmonary disease. 95% UI=95% uncertainty interval.

**Figure 1:**
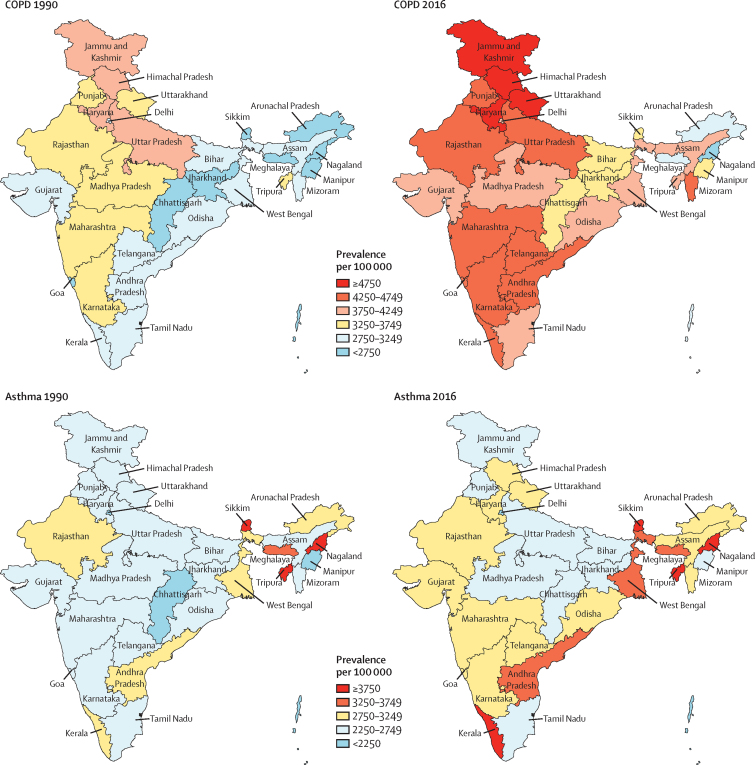
Crude prevalence of COPD and asthma in the states of India, 1990 and 2016 COPD=chronic obstructive pulmonary disease.

The crude prevalence of COPD in India in 2016 was 4·2% (95% UI 4·0–4·4), which was an increase of 29·2% (27·9–30·4) from 3·3 (3·1–3·4) in 1990 (table 2). The crude prevalence increased significantly from 1990 to 2016 in all ETL state groups. In 2016, the crude prevalence of COPD was highest in the high ETL state group, whereas age-standardised prevalence was highest in the low ETL state group. The highest crude COPD prevalence in an individual state was 2·1 times that of the lowest in 2016 (figure 1). The crude prevalence was highest in the contiguous north Indian states of Jammu and Kashmir, Himachal Pradesh, Uttarakhand, and Haryana, which were distributed across lower-middle, higher-middle, and high ETL state groups. The next highest COPD prevalence values were in Rajasthan and Uttar Pradesh (low ETL) and Punjab (high ETL) in the north, Mizoram (lower-middle ETL) in the northeast, and Maharashtra, Goa, Kerala, Karnataka, Andhra Pradesh, and Telangana, which belonged to the higher-middle and high ETL state groups in the west and south of India. The number of COPD cases in India increased from 28·1 million (95% UI 27·0–29·2) in 1990 to 55·3 million (53·1–57·6) in 2016. The number of COPD cases in each state in 2016 is shown in the appendix (p 34).

**Table 2:** Change in prevalence of COPD and asthma in the states of India grouped by epidemiological transition level, 1990 to 2016

	**Crude prevalence per 100 000 (95% UI)**			**Age-standardised prevalence per 100 000 (95% UI)**		
	1990	2016	Percentage change, 1990–2016	1990	2016	Percentage change, 1990–2016
COPD*						
Low ETL (626 million)	3321 (3185 to 3459)	4011 (3843 to 4177)	20·8% (19·3 to 22·2)	6109 (5856 to 6361)	5884 (5640 to 6115)	−3·7% (−4·8 to −2·6)
Lower-middle ETL (92 million)	3144 (3017 to 3272)	4238 (4067 to 4423)	34·8% (33·0 to 36·6)	5909 (5660 to 6166)	5726 (5488 to 5968)	−3·1% (−4·3 to −1·9)
Higher-middle ETL (446 million)	3249 (3118 to 3378)	4397 (4222 to 4580)	35·4% (34·1 to 36·7)	5813 (5576 to 6049)	5433 (5207 to 5656)	−6·5% (−7·4 to −5·7)
High ETL (152 million)	3106 (2979 to 3229)	4409 (4223 to 4591)	41·9% (40·0 to 43·8)	5006 (4801 to 5212)	4656 (4459 to 4847)	−7·0% (−8·1 to −5·7)
India (1316 million)	3254 (3124 to 3385)	4204 (4032 to 4378)	29·2% (27·9 to 30·4)	5823 (5583 to 6061)	5529 (5298 to 5752)	−5·0% (−5·9 to −4·2)
Asthma*						
Low ETL (626 million)	2642 (2480 to 2815)	2744 (2583 to 2915)	3·8% (1·0 to 6·5)	3479 (3277 to 3691)	3352 (3156 to 3563)	−3·7% (−5·7 to −1·7)
Lower-middle ETL (92 million)	2698 (2543 to 2864)	2971 (2809 to 3143)	10·1% (6·9 to 13·4)	3611 (3417 to 3832)	3505 (3307 to 3716)	−2·9% (−5·1 to −0·7)
Higher-middle ETL (446 million)	2693 (2547 to 2854)	3016 (2852 to 3194)	12·0% (9·1 to 15·1)	3540 (3358 to 3740)	3400 (3218 to 3607)	−4·0% (−5·8 to −2·1)
High ETL (152 million)	2532 (2382 to 2689)	2965 (2782 to 3167)	17·1% (13·2 to 20·8)	3206 (3023 to 3400)	3072 (2885 to 3266)	−4·2% (−6·2 to −1·9)
India (1316 million)	2648 (2494 to 2814)	2877 (2715 to 3051)	8·6% (6·1 to 11·4)	3468 (3276 to 3670)	3336 (3146 to 3537)	−3·8% (−5·3 to −2·1)

COPD=chronic obstructive pulmonary disease. ETL=epidemiological transition level. 95% UI=95% uncertainty interval.

* Population in 2016 given in parentheses.

The crude prevalence of asthma in India in 2016 was 2·9% (95% UI 2·7–3·1%), which was similar to the rate in 1990 (table 2). There was no gradient between the ETL state groups for either crude or age-standardised asthma prevalence in 2016, and there was no significant change from 1990 to 2016 for any ETL state group. The ratio of highest to lowest prevalence across the states in India was 2·0 in 2016. Asthma prevalence in 2016 was highest in the northeast states of Sikkim, Tripura, and Nagaland (lower-middle ETL), and in southern state of Kerala (high ETL) (figure 1). The next highest asthma prevalence was in Andhra Pradesh and West Bengal (higher-middle ETL) and in Meghalaya (low ETL). The number of asthma cases in India increased from 22·9 million (21·5–24·3) in 1990 to 37·9 million (35·7–40·2) in 2016. The numbers of asthma cases in each state in 2016 are shown in the appendix (p 34).

**Figure 2:**
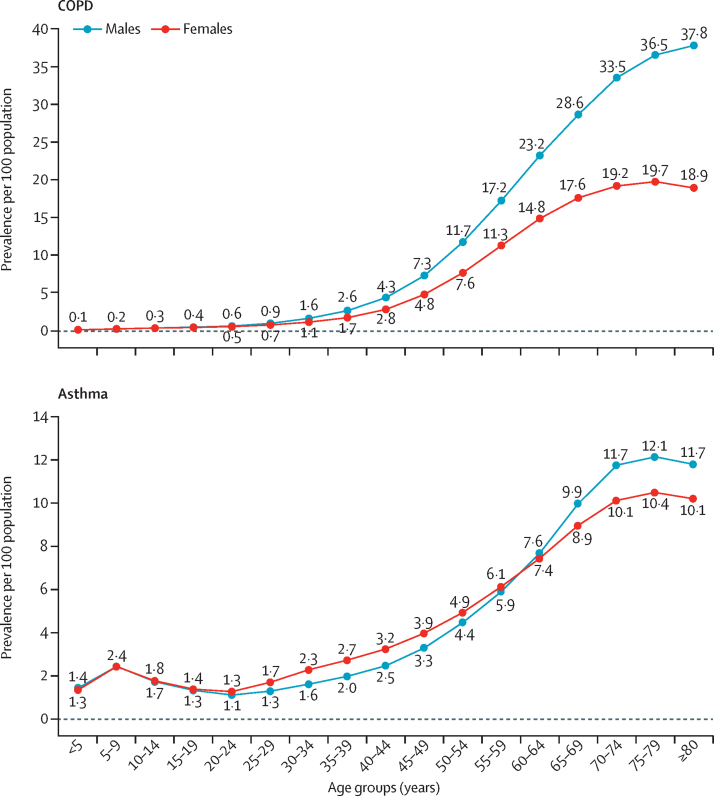
Age-sex-specific prevalence of COPD and asthma in India, 2016 COPD=chronic obstructive pulmonary disease.

The age-specific prevalence of COPD increased rapidly after the age of 30 years, with a greater increase in men than in women, reaching the highest prevalence among men in the 80 years or older age group (37·8%, 95% UI 35·7–40·0) and among women in the 75–79 years age group (19·7%, 18·5–21·0; figure 2; appendix p 35). Asthma prevalence dropped slightly after the age of 9 years and increased after the age of 25 years, reaching the highest prevalence in the 75–79 years age group, at 12·1% (10·7–13·5) among men and 10·4% (9·1–11·9) among women.

**Figure 3:**
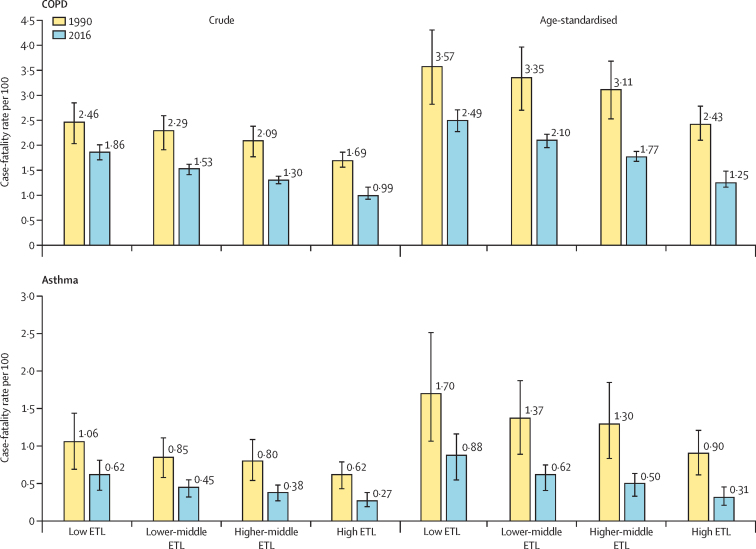
Case-fatality rates for COPD and asthma in the states of India grouped by ETL, 1990 and 2016 Error bars represent 95% uncertainty intervals. COPD=chronic obstructive pulmonary disease. ETL=epidemiological transition level.

The crude case-fatality rate of COPD in India in 2016 was 1·53% (95% UI 1·44–1·63), which was 30·9% (17·2–39·2) less than in 1990 (2·2%, 1·88–2·53; appendix p 36). There was an increasing gradient in the crude and age-standardised COPD case-fatality rate from the high ETL state group to the low ETL state group (figure 3). The reduction of the crude and age-standardised COPD case-fatality rates from 1990 to 2016 was smallest in the low ETL state group (appendix p 36). The crude asthma case-fatality rate in India was 0·48% (0·33–0·62) in 2016, a reduction of 45·9% (31·0–59·0) from the rate in 1990 (0·89%, 0·61–1·22). However, this rate was significantly higher in the low ETL state group than in the high ETL state group in 2016 (figure 3). The case-fatality rate for asthma also had an increasing gradient from the high ETL state group to the low ETL state group. The number of COPD deaths in India increased from 624 000 (508 000–741 000) in 1990 to 848 000 (765 000–939 000) in 2016, while the number of asthma deaths in 2016 was 183 000 (118 000–247 000), which was not significantly different from that in 1990.^21^ The number of COPD and asthma deaths in each state in 2016 are shown in the appendix (p 34).

**Figure 4:**
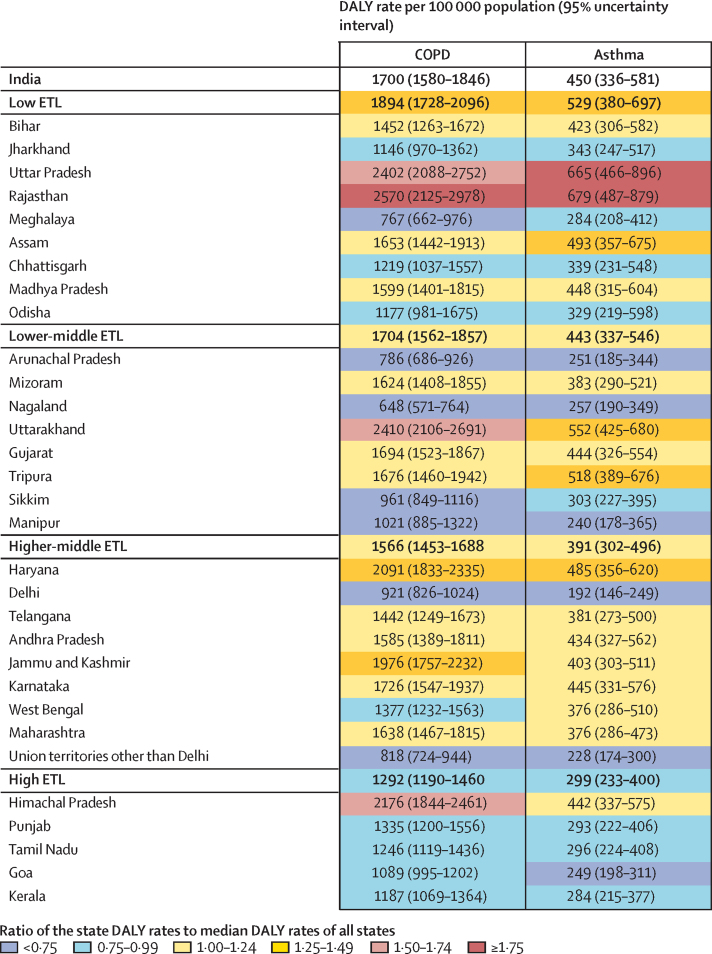
Crude DALY rates due to COPD and asthma in the states of India grouped by epidemiological transition level, 2016 DALY=disability-adjusted life-year. COPD=chronic obstructive pulmonary disease. ETL=epidemiological transition level.

The age-standardised COPD DALY rate in India decreased by 36·2% (95% UI 26·1–42·7) from 1990 to 2016 (appendix p 37). The reduction in crude COPD DALY rate during this period was similar across the ETL state groups, although the reduction in the age-standardised DALY rate was lower in the low ETL and lower-middle ETL state groups. The age-standardised COPD DALY rate in the low ETL state group was 2·1 times higher than that in the high ETL state group. The highest crude COPD DALY rate for an individual state was 4·0 times that of the state with the lowest rate in 2016 (figure 4). The crude rate was highest in the north Indian states of Rajasthan and Uttar Pradesh (low ETL) and Uttarakhand (lower-middle ETL), followed by Himachal Pradesh (high ETL).

The age-standardised asthma DALY rate in India decreased by 54·1% (95% UI 44·0–64·1) from 1990 to 2016 (appendix p 37). The age-standardised DALY rate of asthma in the low ETL state group was 2·4 times higher than that in the high ETL state group. The ratio of the highest asthma DALY rate to the lowest rate across the states of India in 2016 was 3·5 (figure 4). The two large north Indian states of Rajasthan and Uttar Pradesh (both low ETL) had the highest DALY rate due to asthma.

**Figure 5:**
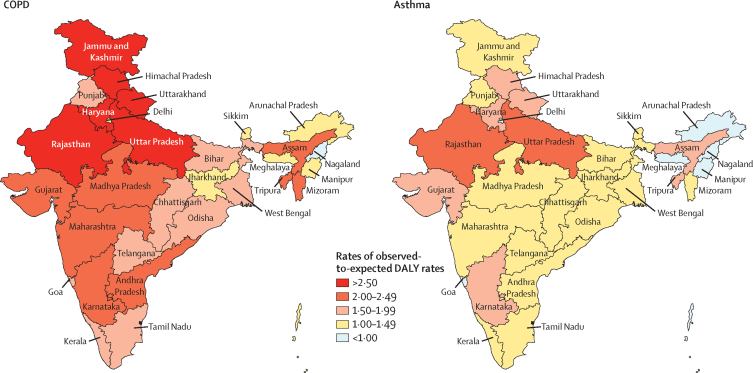
Ratio of observed-to-expected DALY rates due to COPD and asthma in the states of India, 2016 Expected values based on other locations globally at a similar level of Socio-demographic Index. DALY=disability-adjusted life-year. COPD=chronic obstructive pulmonary disease.

In 2016, the age-standardised DALY rate due to COPD in India was 2·3 times higher than that of other locations globally with a similar level of SDI, and the DALY rate of asthma was 1·6 times higher. Every state in India except Nagaland in the northeast had a higher DALY rate due to COPD than the rates globally for their respective levels of SDI, and most states had higher DALY rates of asthma (figure 5). This ratio of the observed-to-expected DALY rates was generally highest in the north Indian states.

**Figure 6:**
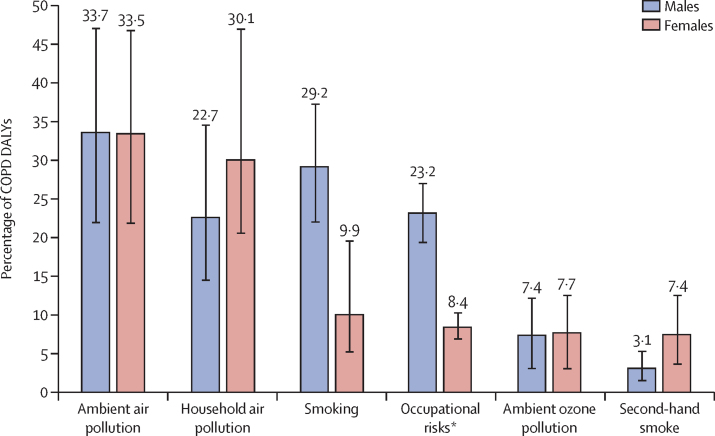
Percentage of COPD DALYs attributable to different risk factors in India by sex, 2016 Error bars represent 95% uncertainty intervals. COPD=chronic obstructive pulmonary disease. DALY=disability-adjusted life-year. *Occupational risks include occupational particulate matter, gases, and fumes, and occupational exposure to second-hand smoke.

Of the COPD DALYs in India in 2016, 53·7% (95% UI 43·1–65·0) were attributed to air pollution, 25·4% (19·5–31·7) to tobacco use, and 16·5% (14·1–19·2) to occupational risks, making these the leading risk factors for COPD. Among the components of these groups of risk factors, 33·6% (21·7–46·7) of COPD DALYs could be attributed to ambient air pollution, 25·8% (16·9–39·8) to household air pollution, and 21·0% (15·4–27·1) to smoking. It is important to note that the cumulative impact of the risk factors would be less than the simple addition of their individual contributions because the risk factors overlap. Additionally, the population attributable fractions of the individual components can add up to more than their sum even if they are independent. The proportional contributions of household air pollution and second-hand smoke to COPD DALYs were greater in women than in men, whereas the contributions of smoking and occupational risks were greater in men (figure 6). The contribution of household air pollution to DALYs due to COPD was larger in the low ETL state group, and there was a decreasing gradient for both ambient and household air pollution going from the low ETL state group to the high ETL state group (appendix p 38). There were wide variations between the states in terms of the relative contributions of the different risk factors for COPD DALYs (appendix p 39). The heterogeneity of air pollution exposure and smoking prevalence across the states of India have been reported elsewhere.^2^, ^3^, ^21^, ^22^

The age-standardised prevalence of COPD in India was 1·5 times the global average prevalence in 2016, and the age-standardised DALYs per person with COPD were 1·7 times the global average (appendix p 40).^20^ The age-standardised prevalence of asthma in India was 0·7 times the global average in 2016, but the age-standardised DALYs per person with asthma were 2·4 times the global average.^20^

## Discussion

Our findings highlight the increasing contribution of chronic respiratory diseases to the disease burden in India over the past quarter century, with COPD now ranking as the second leading individual cause of disease burden in India. With 18% of the global population, India has a disproportionately high 32% of the global DALYs from chronic respiratory diseases.

In 2016, the prevalence of COPD in India was 4·2% and the prevalence of asthma 2·9%. India has a prevalence of COPD that is higher than the global average, as well as more DALYs per person with COPD or asthma than the global average. Most states in India had higher rates of DALYs from COPD and asthma compared with locations elsewhere in the world at similar levels of SDI. However, there were substantial variations between the states. The case-fatality rates for COPD and asthma decreased across all ETL state groups from 1990 to 2016, but these rates were twice as high in the less developed low ETL state group than in the high ETL state group in 2016. The DALY rate due to COPD varied four times between the states, and that of asthma three times. Rajasthan and Uttar Pradesh in north India, both of which are in the low ETL state group, had the highest DALY rates of COPD and asthma in 2016.

From 1990 to 2016, the crude prevalence of COPD in India increased by 29% and that of asthma by 9%. However, age-standardised prevalence declined by 5% for COPD and 4% for asthma. This finding suggests that the overall increases in prevalence are mainly due to ageing of the population in India. Understanding changes in the risk factors for COPD, which makes up three-quarters of the chronic respiratory disease burden in India, would be instructive for placing the changes in prevalence in perspective. The dominant risk factor for COPD in India in 2016 was air pollution, which contributed more than half the DALYs due to COPD, followed by smoking, which contributed a quarter of the COPD DALYs. As described previously by the India State-Level Disease Burden Initiative, ambient air pollution exposure has increased in most parts of India since 1990, whereas exposure to household air pollution has decreased because of a reduction in the use of solid fuels, although it is still quite high.^2^, ^3^ The prevalence of smoking has also decreased in all parts of India since 1990, as reported in an accompanying Article.^22^ The mixed pattern of changes in these major risk factors for COPD are likely to have played a part in the small change in age-standardised prevalence of COPD from 1990 to 2016, although there is likely to be a range of other factors also that are contributing to this trend. There have been increasing attempts in India to understand the association between causes other than smoking and COPD, given that a substantial portion of COPD cases occur in people who have never smoked.^23^ Findings from studies in India have identified associations between chronic respiratory diseases and non-smoking-related factors, such as outdoor air pollution from particulate matter, indoor air pollution from biomass fuels, occupational exposure to crop dust, dust from mines, chemicals, poor socioeconomic status, poor nutrition, overcrowding, and residence in urban slums.^23^, ^24^, ^25^ From a global perspective, the proportion of COPD DALYs attributable to smoking in India is smaller than the global average in 2016, whereas the proportion of COPD DALYs attributable to air pollution is larger.^20^

Despite declines in crude and age-standardised DALY rates of COPD and asthma, suggesting improvements in health care, India still has substantially higher DALYs per person with COPD and asthma than the global average. This high rate of health loss from COPD is due to issues related to its relatively late diagnosis and management, even with the improvements in health care over time in India. Evidence suggests that COPD is underdiagnosed in India, partly because diagnosis is mostly based on symptoms rather than spirometry and also because symptomatic people are often late in seeking care because of insufficient awareness of the disease.^26^, ^27^ The prescription of inhalation devices, which is often needed to manage the late stages of COPD, is often perceived as a stigma in rural areas.^27^ Most symptomatic individuals tend to seek treatment from the non-formal health practitioners of alternative medicines and faith healing, who often prescribe harmful and toxic agents. Women at the early stages of the disease are less symptomatic than men, which, combined with their less common health-seeking behaviour, poses additional challenges to COPD diagnosis.^28^

The larger than average disease burden per person with asthma in India indicates poor management of this disease in India. This finding is consistent with results from a multicentre study done in several different parts of the world, which showed that asthma management was worst in India among the Asia-Pacific countries studied.^29^ Unlike COPD, which is mostly associated with ageing, asthma affects all age groups including school-going children. Inadequate clinical knowledge of asthma among practising physicians, poor utilisation of diagnostic tools for asthma, limited use of inhaled medications, and the existence of several misconceptions and misbeliefs in the community seem to be the likely contributing factors to the high DALY rate per person with asthma in India.^29^, ^30^, ^31^ Studies have suggested that poor management of asthma in childhood also affects lung growth and increases the risk of developing COPD at later ages.^32^ Another challenge in the adequate management of asthma is the misclassification of asthma as COPD, and vice versa, because their clinical features overlap. Furthermore, an asthma–COPD overlap syndrome has been described in which patients have mixed features of asthma and COPD with air flow limitation.^33^

The National Programme for Prevention and Control of Cancer, Diabetes, Cardiovascular Diseases and Stroke was launched in 2010 and a component for chronic respiratory diseases was added later.^34^ However, this respiratory disease component represents a small part of the programme, involving only referrals of suspected cases to the district hospitals. Important components, such as health promotion, early diagnosis, and management, are lost in the mix of other non-communicable diseases such as cardiovascular disease and diabetes. There have been calls for a national programme on the control of COPD and other chronic respiratory diseases in India so that these diseases can receive attention commensurate with their contribution to the disease burden.^26^ A task force of India's health ministry that focuses on comprehensive primary health-care rollout proposed in 2014 that health promotion activities, screening, and management guidelines for COPD, asthma, and pneumoconiosis be included in the service packages, in addition to other non-communicable diseases.^35^ These activities related to chronic respiratory diseases are yet to be implemented on a large scale. There are some examples of efforts by the public sector towards the early detection and management of COPD, as in the state of Kerala.^36^ However, much more extensive efforts are needed in India to improve early detection and management of COPD and asthma. These further efforts come in addition to the need for adequate measures to reduce exposure to risk factors for these major chronic respiratory diseases. Ongoing efforts to control smoking and reduce household solid fuel use are having positive effects, and these need to be sustained and enhanced.^2^, ^3^, ^22^, ^37^, ^38^ Additionally, huge multisectoral efforts are needed to reduce the high level of exposure to ambient air pollution in all parts of India, especially in the northern states.^2^, ^3^

The general limitations of the GBD methods,^12^, ^13^, ^14^, ^15^, ^16^ and those for chronic respiratory diseases, have been discussed previously.^1^ A limitation specific to India in ascertaining the causes of death is an incomplete reporting system for medically certified causes of death, which only covers a small proportion of deaths and has variable coverage between states. This limitation is partly addressed by the inclusion of population-based verbal autopsy cause-of-death data from the Sample Registration System, which covers every state.^2^ Data on COPD and asthma morbidity and sequelae have been reported from some states as part of the BOLD, INSEARCH, and ISAAC studies,^17^, ^18^, ^19^ but are not available for many states. When data are scarce, GBD uses covariates with known associations with the outcome of interest to arrive at the best possible estimates. More efforts are needed in India to understand the natural history of COPD and asthma in different parts of the country. It would also be useful to develop occupational health registries to better understand the contribution of occupational exposures to chronic respiratory diseases. The strengths of the findings presented in this report are the use of standardised GBD methodology and the inclusion of all accessible data from multiple sources, as well as the substantial contributions from a large network of experts from India in the analysis and interpretation of the findings.

In conclusion, a third of the total global health loss from chronic respiratory diseases occurs in India. There is marked heterogeneity between the states of India in this burden and the associated risks, highlighting the need for individual states to adopt different policy approaches according to the trajectory of the disease burden they are facing. The almost negligible large-scale effort to address major chronic respiratory diseases at the population level in India must be improved rapidly to reduce the current disproportionately high health loss from these diseases across India.
