# Supplementary material for: The burden of chronic respiratory diseases and their heterogeneity across the states of India: the Global Burden of Disease Study 1990–2016
Source: Lancet Glob Health. 2018 Sep 12;6(12):e1363–74. doi: 10.1016/S2214-109X(18)30409-1 (PMC6227385; doi:10.1016/S2214-109X(18)30409-1)
Supplement: Supplementary appendix [file mmc1.pdf]

THE LANCET
Global Health

Supplementary appendix

This appendix formed part of the original submission and has been peer reviewed. We post it as supplied by the authors.

Supplement to: India State-Level Disease Burden Initiative CRD Collaborators. The burden of chronic respiratory diseases and their heterogeneity across the states of India: the Global Burden of Disease Study 1990–2016. *Lancet Glob Health* 2018; published online Sept 12. http://dx.doi.org/10.1016/S2214-109X(18)30409-1.

The burden of chronic respiratory diseases and their heterogeneity across the states of India: the Global Burden of Disease Study 1990–2016

India State-Level Disease Burden Initiative CRD Collaborators

Web Appendix

Correspondence to: Prof. Lalit Dandona, lalit.dandona@phfi.org

Table of Contents

1. GBD 2016 chronic respiratory diseases burden estimation methods ... 3
2. GBD 2016 India data inputs for chronic respiratory diseases mortality, morbidity, risk factors, and covariates ... 23
3. Prevalent cases of and deaths from COPD and asthma in the states of India, 2016 ... 34
4. Age-sex-specific prevalence of COPD and asthma in India, 2016 ... 35
5. Change in case-fatality rates of COPD and asthma in the states of India grouped by epidemiological transition level, 1990 to 2016 ... 36
6. Change in DALY rates of COPD and asthma in the states of India grouped by epidemiological transition level, 1990 to 2016 ... 37
7. Percent of COPD DALYs attributable to risk factors in the states of India grouped by epidemiological transition level, 2016 ... 38
8. Percent of COPD DALYs attributable to risk factors in the states of India, 2016 ... 39
9. Comparison of age-standardised prevalence and DALYs per case of COPD and asthma in India versus the global average, 2016 ... 40

1. GBD 2016 chronic respiratory diseases burden estimation methods

The material presented here is adapted from the following sources:

- GBD 2016 Disease and Injury Incidence and Prevalence Collaborators. Global, regional, and national incidence, prevalence, and years lived with disability for 328 diseases and injuries for 195 countries, 1990–2016: a systematic analysis for the Global Burden of Disease Study 2016. Lancet 2017; 390: 1211–59.
- GBD 2016 Causes of Death Collaborators. Global, regional, and national age-sex specific mortality for 264 causes of death, 1980–2016: a systematic analysis for the Global Burden of Disease Study 2016. Lancet 2017; 390: 1151–210.
- GBD 2016 Risk Factors Collaborators. Global, regional, and national comparative risk assessment of 84 behavioural, environmental and occupational, and metabolic risks or clusters of risks, 1990–2016: a systematic analysis for the Global Burden of Disease Study 2016. Lancet 2017; 390: 1345–422.
- Cohen AJ, Brauer M, Burnett R, et al. Estimates and 25-year trends of the global burden of disease attributable to ambient air pollution: an analysis of data from the Global Burden of Diseases Study 2015. Lancet 2017; 389: 1907–18.

The GBD cause list is organised hierarchically into four levels. At each level of the hierarchy, the set of causes is mutually exclusive and collectively exhaustive. Levels 1 and 2 represent general groupings. The broad group "chronic respiratory diseases" is at level 2 under the level 1 group "non-communicable diseases". Level 3 includes five chronic respiratory diseases (CRD) groups: chronic obstructive pulmonary disease, pneumoconiosis, asthma, interstitial lung disease and pulmonary sarcoidosis, and other chronic respiratory diseases. Level 4 includes four groups, all under the parent level 3 "pneumoconiosis" group: silicosis, asbestosis, coal worker's pneumoconiosis and other pneumoconiosis.

A. GBD case definitions of chronic respiratory diseases

Chronic obstructive pulmonary disease (COPD)

COPD was defined as in the Global Initiative for Chronic Obstructive Lung Disease (GOLD) classification: a measurement of <0.7 FEV1/FVC (FEV1 is forced expiratory value in one second, and FVC is forced vital capacity) on spirometry after bronchodilation. The definitions of the severity classes in the GOLD classification are provided below.

GOLD class	FEV1 score
I: Mild	>=80% of normal
II: Moderate	50-79% of normal
IV: Severe	<50% of normal

Asthma

Asthma was defined as a chronic lung disease marked by spasms in the bronchi usually resulting from an allergic reaction or hypersensitivity and causing difficulty in breathing. It was reported as a doctor's diagnosis and wheezing in the past year.

Pneumoconiosis

Pneumoconiosis was defined as a chronic lung disease typified by lung scarring and other interstitial damage caused by exposure to dust and other containments, usually through occupational exposure.

Interstitial lung disease and pulmonary sarcoidosis

Interstitial lung diseases and pulmonary sarcoidosis were defined as a collection of chronic respiratory diseases that impair lung function and oxygen uptake through scarring and/or inflammation. For interstitial lung disease, GBD uses the American Thoracic Society as the gold standard definition.

Other chronic respiratory diseases

This is a residual category with no single case definition.

B. List of ICD codes mapped to the GBD cause list

The codes used by GBD Study 2016 from the 9th and 10th revisions of the International Statistical Classification of Diseases and Related Health Problems (ICD) are listed below:

Cause	ICD10	ICD9
Chronic obstructive pulmonary disease	J40, J41, J42, J43, J44, J47	490-492, 494, 496
Asthma	J45, J46	493
Coal worker's pneumoconiosis	J60	500
Silicosis	J62	501
Asbestosis	J61	502
Other pneumoconiosis	J63	503, 504
Unspecified pneumoconiosis	J64	505
Interstitial lung diseases	J84	
Pulmonary sarcoidosis	D86	135

C. GBD data and analysis framework

The overview of data inputs and analysis framework for GBD is shown in the following flowchart:

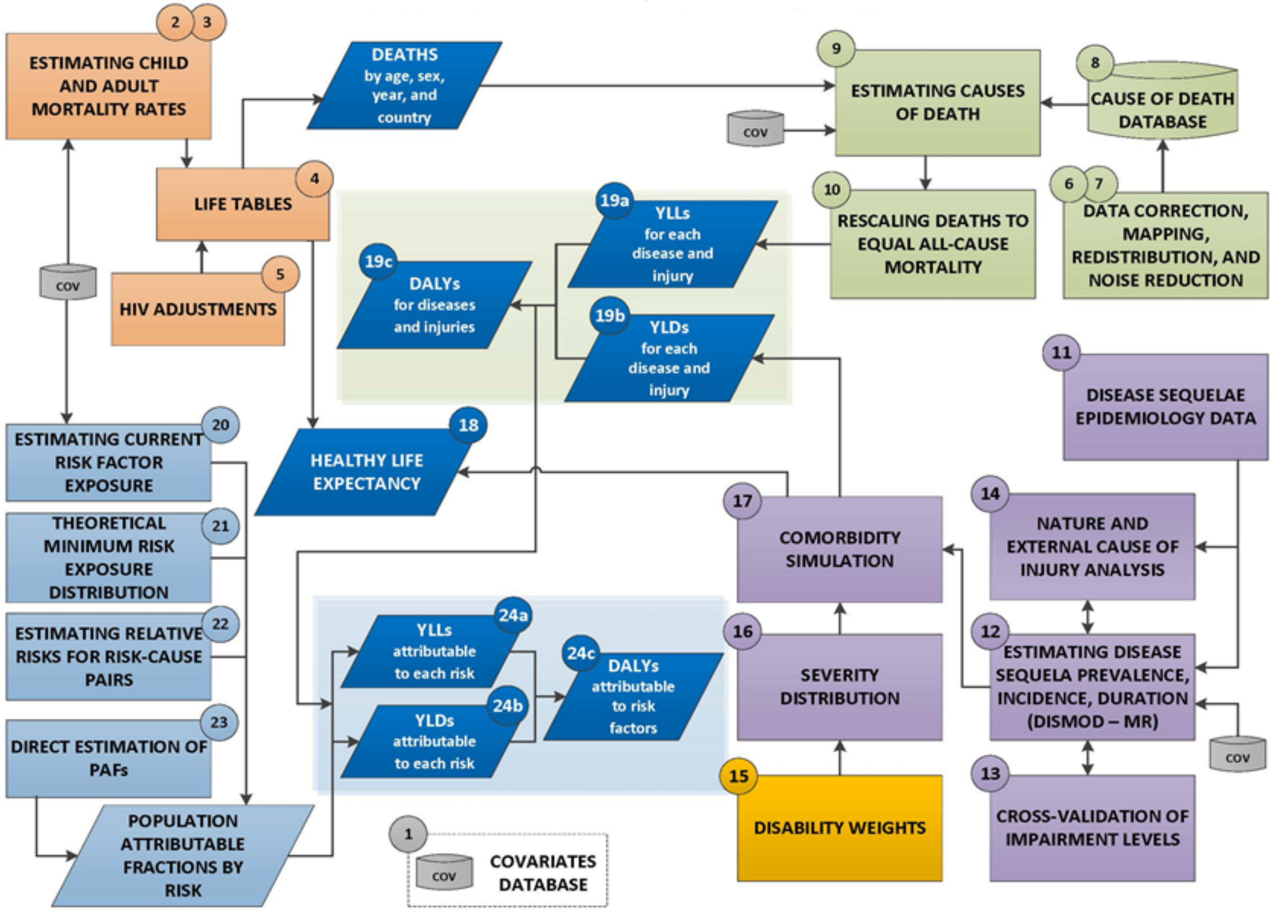

YLLs is years of life lost. YLDs is years lived with disability. DALYs is disability-adjusted life-years. PAFs is population attributable fractions. Rectangular boxes represent analytical steps, cylinders represent databases, and parallelograms represent intermediate and final results.

The flowchart above illustrates the flow of the key components of the GBD estimation process, including:

1. Incorporation of appropriate covariates (step 1)
2. All-cause mortality estimation (steps 2-5): the data come from sources such as censuses, surveys and vital registrations. The all-cause mortality estimation process (steps 2-4) can be divided into four distinct but interconnected areas: child mortality and adult mortality between ages 15 and 60, estimation of a complete set of age-specific death rates, estimation of HIV mortality and final estimates of age-specific mortality including HIV and fatal discontinuities (also known as mortality shocks) (step 5).
3. Causes of death estimation (steps 6-9): cause of death data are derived from vital registrations, verbal autopsy studies, mortality surveillance and, for selected causes, police records, crime reports and data collection systems for deaths due to conflict and natural disasters (step 7). Extensive data corrections and redistributions of ill-defined causes are made to correct for measurement bias between data sources. Cause of death ensemble modelling (CODEm), an ensemble model, is a systematized approach to analysing cause of death data for all but a few causes (step 9). CODEm explores a wide range of modelling approaches and varying predictive covariates to find an ensemble of best-performing models based on statistical tests. To do so, 30% of the data are withheld from each model and the model fit is evaluated by how well it covers the data that were left out. By repeating this process many times over the best performing models are selected. As all results in GBD are estimated 1,000 times over to propagate all sources of uncertainty, we end up with an ensemble of up to 100 or more different types of models and covariates that are selected among the 1,000 runs.
4. Rescaling deaths to equal all-cause mortality (step 10): as all these estimates are made separately for each disease and injury, the sum of these could exceed or fall below the all-cause mortality estimated from the demographic analyses of steps 2 to 5. Therefore, we rescale all deaths by age, sex, geography, year and cause to match the all-cause death estimates (this process is called CoDCorrect).
5. Estimation of disease sequelae prevalence, incidence, and duration (steps 11-12): population surveys, cohort studies, administrative records of hospitalisations and other health service encounters, disease registries, notifications, surveillance systems are the main data sources for non-fatal estimation (step 11). Extensive corrections of data to deal with measurement bias arising from study design or case definitions are applied. DisMod-MR 2.1 is the main analytical tool for non-fatal estimation (step 12). It is a Bayesian meta-regression software program that uses a lognormal model. The meta-regression component allows corrections for known sources of measurement error. Its core function is to make estimates of prevalence and incidence of disease that are consistent with data on mortality risk and remission (defined in GBD as the 'cure rate'). For a select number of causes that do not fit well in the three state model (alive without disease, prevalent case of disease and death) of DisMod-MR 2.1 we use alternative modelling strategies.
6. Cross-validation of impairment levels (step 13): for a number of impairments in GBD terminology, such as anaemia, heart failure, hearing and vision loss, we first estimate the total levels of prevalence and incidence and then ensure that all sequelae of diseases that lead to this impairment add up to the total.
7. Analysis of the nature and external cause of injury is done separately (step 14).
8. Assignment of severity distributions for the main disabling conditions (step 15): in GBD terminology sequelae are the disabling consequences for which we make estimates. All sequelae are defined to be mutually exclusive and collectively exhaustive. Many diseases have sequelae with a gradation by severity such as mild, moderate and severe dementia. Often the epidemiological data on severity distribution is sparse. Therefore, we first model the epidemiology of all cases of disease and then apply a severity distribution from the sparser data.
9. Assignment of disability weights for health states (step 16): each sequela is matched with a health state or combination of health states for which we have a disability weight which quantifies the relative severity. Disability weights were derived from population and internet surveys of over 60,000 respondents answering pair-wise comparison question of random combinations of health states. Each pair of health states was described with brief lay descriptions highlighting the main symptoms and impairments. Respondents were asked to nominate the 'healthier' of each presented pair. Analytical methods exist to formalise the intuition that if the majority of respondents nominate one health state in a pair as the healthier these lie farther apart on a severity scale than pairs assigned similar proportions as the healthier. In order to anchor estimates on a 0-1 scale of severity, a subset of respondents was asked additional population health equivalence questions on a selection of health states. These questions ask for a choice of the greater amount of health produce by two health programs; one that prevented sudden death in 1,000 persons and another that prevented the onset of a GBD health state for the rest of 2,000, 5,000 or 10,000 persons' lives.
10. Simulation of comorbidity (step 17): the last step of non-fatal estimation is a microsimulation ('COMO") to deal with comorbidity. For every age, sex, geography and year, 40,000 hypothetical persons are generated who have none, one or more of the GBD sequelae. In those with multiple sequelae their combined level of disability is estimated multiplicatively. That means we assume the disability from having two health states is less than the sum of the corresponding disability weights. This avoids assigning disability greater than one to any individual which would indicate that person is worse off than being dead.

11. Estimation of healthy life expectancy (step 18): health life expectancy is estimated from the life tables generated in step 4 and the all-cause YLD rates from step 19b.
12. Computation of YLLs, YLDs, and DALYs from diseases and injuries with uncertainty (steps 19a-19c): YLLs (step 19a) are estimated as the product of counts of death by ages, sex, geography, year and cause and a normative life expectancy at the age of the death. The GBD standard life expectancy used as this norm is a compilation of the lowest observed mortality rates by age in all mortality data collections of populations greater than 5 million. The standard life table reflects a life expectancy at birth of 86.59 years. YLDs are the output from COMO (step 19b). DALYs are the simple addition of YLLs and YLDs (step 19c).
13. Risk factor estimation (steps 20-24): GBD 2016 also makes estimates for individual and combined risk factors. This involves estimation of risk factor exposure (step 20); the formulation of a minimum level of exposure to each risk that is associated with the least amount of health loss (step 21); derivation of relative risks of disease outcomes for each pair of a risk factor and a disease or injury for which there is judged to be sufficient evidence of a causal relationship (step 22); and the estimation of population attributable fractions of disease caused by each risk factor. For a few risk-outcome pairs it is hard to define exposure and a corresponding risk while directly observed proportions of disease are available, such as for the proportion of HIV/AIDS due to unsafe sex or injecting drug use (step 23). For combinations of risks we assess how much of the risk is mediated through other risks (step 24). For instance, all of the effect of high salt intake is mediated through elevated blood pressure and part of the risk of increased body mass index is through elevated blood pressure, cholesterol or fasting plasma glucose.
14. Computation of YLLs, YLDs, and DALYs attributable to risk factors (steps 25a-25c): YLLs, YLDs and DALYs attributable to each risk factor are generated by multiplying population attributable fractions with disease estimates (steps 25a-c).

D. Chronic respiratory diseases morbidity estimation

Morbidity from chronic respiratory diseases was modelled using the DisMod-MR 2.1 platform. Morbidity estimation for the major chronic respiratory diseases presented in this paper, chronic obstructive pulmonary disease and asthma, are described below. Modelling methods for the remaining chronic respiratory diseases are available in the GBD 2016 non-fatal capstone paper (Lancet 2017; 390: 1211–59).

D.1. Chronic obstructive pulmonary disease (COPD)

The steps in the estimation of non-fatal chronic obstructive pulmonary disease burden or morbidity are shown in the following flowchart:

Chronic Obstructive Pulmonary Disease (COPD)

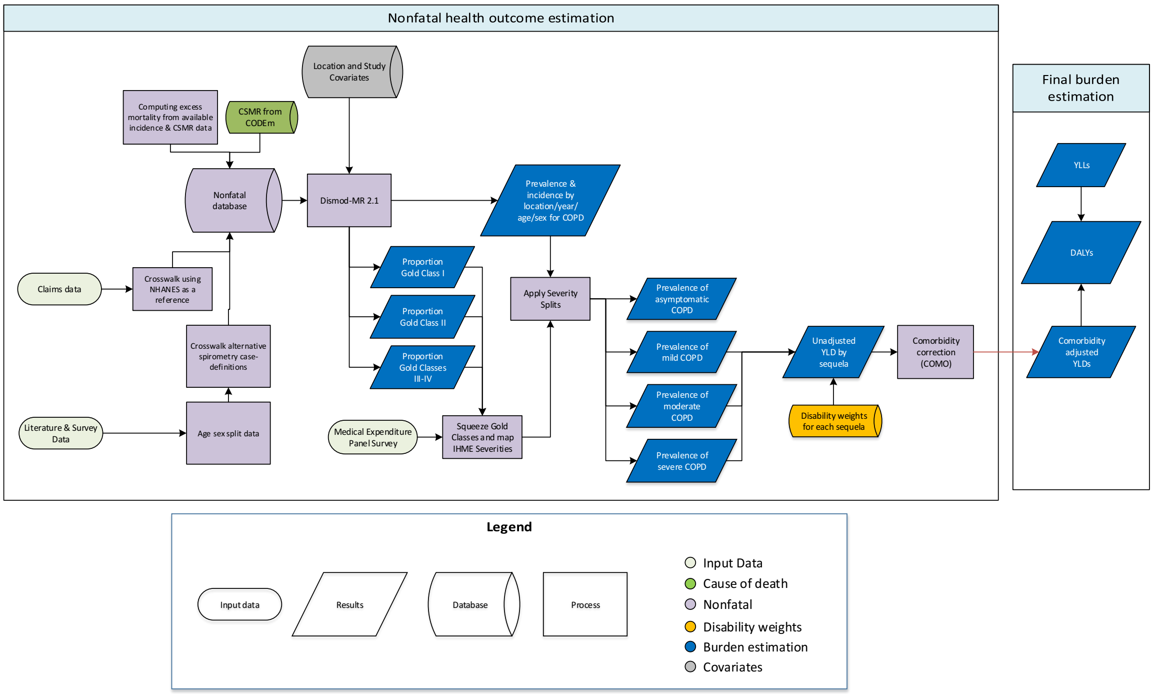

Data

A major data source for COPD morbidity estimation in India was the Burden of Obstructive Lung Disease Initiative Survey (BOLD). Other data sources included population-representative surveys and cohort studies including published and unpublished studies. We included survey data with spirometry measurements. Data using alternative case-definitions of COPD prevalence (i.e. lower limit of normal) were crosswalked to the reference case-definition with age-specific ratios derived from studies reporting prevalence using both the alternative and reference case-definitions. Furthermore, claims data were included if available. Briefly, we determined estimates of COPD prevalence from a database of individual level ICD-coded health service encounters. Persons with any claim associated with COPD were marked as a prevalent case for that year. An age-specific crosswalk was also derived to adjust claims data since it was assumed that survey data with spirometry measurements were more accurate.

Modelling strategy

The estimation of COPD burden occurs in three main steps. First is the estimation of prevalence and incidence using a DisMod-MR 2.1 model. Second is the separate estimation of the proportions by three GOLD class groupings in DisMod-MR 2.1. Third is the combination of these two processes to derive prevalence by severity.

Step 1: Main COPD model
Prior settings include remission of 0 and an incidence ceiling of 0.0002 before age 20. The latter was necessary to avoid a kick-up of estimates in childhood at an age range with few or no primary data.

Claims data for 2000 and 2010 were adjusted via study covariates to account for systematically low estimates relative to the 2012 claims data. Implicit in this adjustment is the assumption that variation between years of claims data is a function of data collection inconsistencies.

We included estimates of cause-specific mortality rate (CSMR) and derived estimates of excess mortality rate (EMR) by dividing every prevalence data point by the CSMR value for the corresponding location, age, sex, and year. We did not estimate EMR for data points with an age range greater than 20 years.

To assist estimation, each model included a series of country-level covariates that describe spatiotemporal patterns. Where available, we used the COPD standardised exposure variables (SEV), which aggregate multiple risk factors into a single variable. We also used the log of lag-distributed income (LDI) on EMR to capture country-level variation of EMR, assuming a negative coefficient (i.e., lower mortality with rising GDP).

Step 2: GOLD class models
The GOLD class models use data from surveys that specified prevalence by GOLD class after expressing the values as a proportion of all COPD cases. We used fixed effects from the SEV scalar and the log of LDI per capita to assist estimation. Coefficients for the covariates used in the modelling for fixed effects are listed below:

Cause	Variable name	Measure	Beta	Exponentiated
COPD	LDI (I$ per capita)	Excess mortality rate	-0.5 (-0.5 — -0.5)	0.61 (0.60 — 0.61)
COPD	Log age-standardised SEV scalar: COPD	Prevalence	0.75 (0.75 — 0.76)	2.12 (2.12 — 2.15)
GOLD I proportion	Socio-demographic Index	Proportion	0.93 (-0.96 — 1.97)	2.54 (0.38 — 7.18)
GOLD I proportion	Log age-standardised SEV scalar: COPD	Proportion	-0.17 (-0.62 — 0.33)	0.84 (0.54 — 1.39)
GOLD II proportion	Socio-demographic Index	Proportion	0.93 (-0.96 — 1.97)	2.54 (0.38 — 7.18)
GOLD II proportion	Log age-standardised SEV scalar: COPD	Proportion	-0.17 (-0.62 — 0.33)	0.84 (0.54 — 1.39)
GOLD III+IV proportion	Socio-demographic Index	Proportion	0.35 (-1.66 — 1.88)	1.42 (0.19 - 6.57)
GOLD III+IV proportion	Log age-standardised SEV scalar: COPD	Proportion	0.018 (-0.8 — 0.78)	1.02 (0.45 — 2.17)

Step 3: Prevalence by severity
The three GOLD class groupings reflect a grading based on a physiological measurement rather than a direct measurement of disease severity. In order to map the epidemiological findings by GOLD Class into the three COPD health states for which we have disability weights, we used the 2001–2011 Medical Expenditure Panel Survey (MEPS) data from the United States. Specifically, we converted the GOLD class designations estimated for the USA in 2005 (the midpoint of MEPS years of analyses) into GBD classifications of asymptomatic, mild, moderate, and severe COPD. The proportion of MEPS respondents reporting any health service contact in the past year for COPD was determined, along with a disability weights value attributable to COPD of 0, mild range (0 to midpoint between disability weights for mild and moderate), moderate range (midpoint of disability weights values mild and moderate to midpoint of disability weights values for moderate and severe) and severe range (midpoint between disability weights values moderate and severe or higher). The algorithm to translate GOLD Class to COPD disability weights categories first assigned GOLD III&IV to severe COPD and what remains to moderate. Next, GOLD class I was assigned to the asymptomatic category first and what remains goes to mild COPD. This algorithm was repeated for each age and sex category and for all 1,000 draws from the DisMod models of GOLD classes and the MEPS analyses. We ended up with proportions of each of the GOLD class categories that map onto GBD COPD health states with uncertainty bounds determined by the 25th and 975th values of the 1,000 draws. These values were then applied to the estimates of the proportion of cases by GOLD class category, after scaling to 100%, by location, year, age, and sex.

The disability weights value for COPD was derived from a regression with indicator variables for all health states reported by MEPS respondents and their reported overall level of disability derived from a conversion of 12-item short form surveys (SF-12) answers to GBD disability weights values. This analysis gave the severity distribution for each GBD cause reported in MEPS after correcting for any comorbid causes individual respondents reported during a year. The table below shows the three health states of COPD and the corresponding lay descriptions and disability weights:

**Health state**	**Lay description**	**Disability weights (95% CI)**
Mild COPD	This person has cough and shortness of breath after heavy physical activity, but is able to walk long distances and climb stairs.	0.019 (0.011–0.033)
Moderate COPD	This person has cough, wheezing, and shortness of breath, even after light physical activity. The person feels tired and can walk only short distances or climb only a few stairs.	0.225 (0.153–0.31)
Severe COPD	This person has cough, wheezing, and shortness of breath all the time. The person has great difficulty walking even short distances or climbing any stairs, feels tired when at rest, and is anxious.	0.408 (0.273–0.556)

D.2. Asthma

The steps in the estimation of non-fatal asthma burden or morbidity are shown in the following flowchart:

Asthma

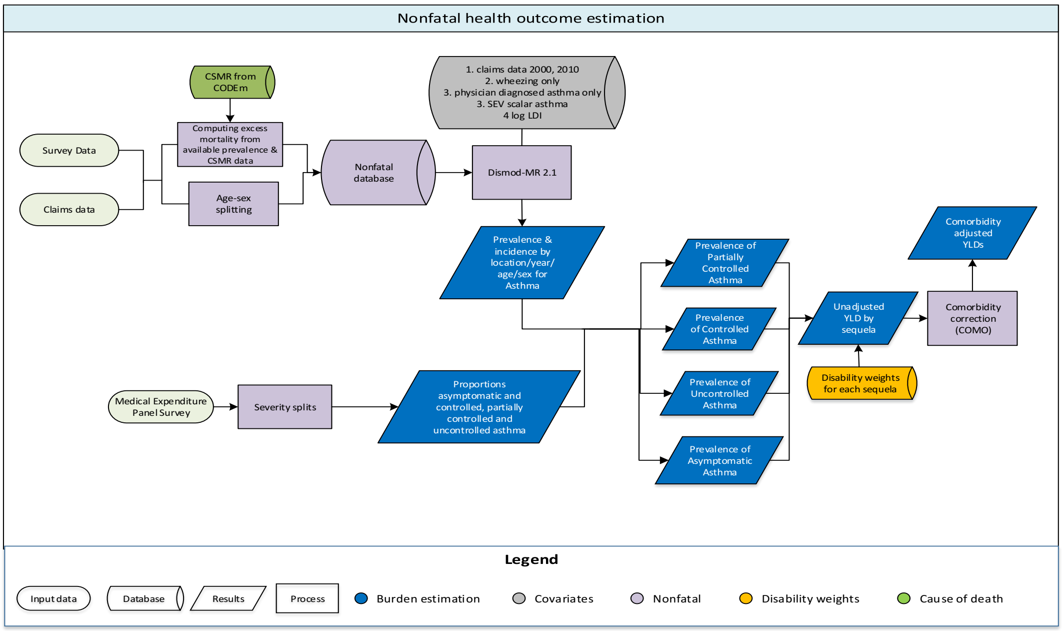

Data

The major data inputs for asthma morbidity in India were International Study of Asthma and Allergies in Childhood (ISAAC); National Family Health Survey; WHO Study on global AGEing and adult health (SAGE); and Indian Study on Epidemiology of Asthma, Respiratory Symptoms and Chronic Bronchitis (INSEARCH). Surveys carried out as part of the ISAAC collaboration were the most important source of prevalence data in children.

In addition to literature and survey data, we used claims data to determine estimates of asthma prevalence from a database of individual level ICD-coded health service encounters for three years. Persons with any claim associated with asthma were marked as a prevalent case for that year. Aggregated estimates were then adjusted using a noise reduction algorithm. These corrected data were then used in the modelling process.

Modelling strategy

We used DisMod-MR 2.1 as the main modelling tool for asthma. Prior settings include a maximum remission of 0.3 (reflecting the upper bound of the highest observed data) and no incidence between the ages of 0 and 0.5 year, as a diagnosis cannot be made in young infants.

Data points from the ISAAC studies were reported for both sexes combined. We sex-split before modelling using the ratios derived from claims data.

Data that describe wheezing in the past year, but do not report presence/absence of an accompanying diagnosis were crosswalked to the reference category using a study-level covariate in DisMod. Studies that only report wheezing were systematically higher than reference data points and were adjusted down by dividing with the exponentiated coefficient. Data that describe prevalence of lifetime diagnosis of asthma but not accompanying wheezing in the past year were also crosswalked to the reference category using a study-level covariate.

To account for country-level differences in excess mortality as a function of available medical care, we used log LDI as a covariate and assumed a negative coefficient.

Claims data for 2000 and 2010 were adjusted via study covariates to account for systematically lower estimates relative to the 2012 claims data. Implicit in this adjustment was the assumption that variation between years of claims data was a function of data-collection inconsistencies.

We included estimates of cause-specific mortality rate (CSMR) and excess mortality rate (EMR) derived as a matched value for each prevalence data point dividing CSMR by prevalence. We restricted these EMR calculations to data points of 20-year age span or less.

To assist estimation, the model includes a series of country-level covariates that describe spatiotemporal patterns. Specifically, we used log LDI and the asthma standardised exposure variable (SEV), a scalar that combines exposure of all GBD risks that influence asthma. A full covariate list, including the study-level covariates, described above are presented in the following table with their associated effects:

Variable name	Measure	Beta	Exponentiated
Wheezing	Prevalence	0.46 (0.43 – 0.50)	1.59 (1.53 — 1.65)
Physician diagnosed asthma only	Prevalence	-0.091 (-0.14 – -0.038)	0.91 (0.87 — 0.96)
Claims data 2000	Prevalence	-0.53 (-0.56 – -0.50)	0.59 (0.57 — 0.61)
Claims data 2010	Prevalence	-0.13 (-0.15 – -0.10)	0.88 (0.86 — 0.90)
Log SEV scalar: asthma	Prevalence	1.24 (1.20 — 1.25)	3.44 (3.33 — 3.49)
Log LDI (I$ per capita)	Excess mortality rate	-0.5 (-0.5 — -0.5)	0.61 (0.61 — 0.61)

The distribution between the three health states is derived from an analysis of MEPS data. Briefly, MEPS is an ongoing survey of health service encounters with as its main objective to collect data on health expenditure. Panels are recruited every year and followed up for a period of two years. Diagnostic information provided by respondents on the reasons for any health care contact are coded into three digit ICD-9 codes by professional coders.

Twice over the two-year follow-up period, respondents are asked to fill in SF-12. From convenience samples asking respondents to fill in SF-12 for 60 of the GBD health states, a mapping has been created from SF-12 scores to GBD disability weights. We performed a regression with indicator variables for all GBD causes that we can identify from the ICD codes in MEPS to derive for each individual with a diagnosis the amount of disability that can be attributed to that condition after controlling for any comorbid conditions. Anyone with a diagnosis of asthma in whom the disability assigned to asthma is negative or zero we assumed is asymptomatic (at the time of asking SF-12 question relating to their health status in the past four weeks). Non-zero values were bin into the three health states assuming a split between these at the midpoint between disability weights values. The table below gives the proportions in MEPS in each of the health states and an asymptomatic state:

**Severity level**	**Lay description**	**Disability weights (95% CI)**	**Severity distribution**
Asymptomatic			36.2% (35.0–37.3%)
Controlled	This person has wheezing and cough once a month, which does not cause difficulty with daily activities.	0.015 (0.007–0.026)	19.9% (13.6–27.8%)
Partially controlled	This person has wheezing and cough once a week, which causes some difficulty with daily activities.	0.036 (0.022–0.055)	20.6% (15.1–25.8%)
Uncontrolled	This person has wheezing, cough, and shortness of breath more than twice a week, which causes difficulty with daily activities and sometimes wakes the person at night.	0.133 (0.086–0.192)	23.3% (18.7–30.3%)

E. Chronic respiratory diseases mortality estimation

Mortality estimation and modelling methods for the major chronic respiratory diseases presented in this paper (chronic obstructive pulmonary disease and asthma) are described below. Details about the other chronic respiratory diseases are available in the GBD 2016 cause of death capstone paper (Lancet 2017; 390: 1151–210).

The approach to cause of death estimation for chronic respiratory diseases is shown in the following flowchart:

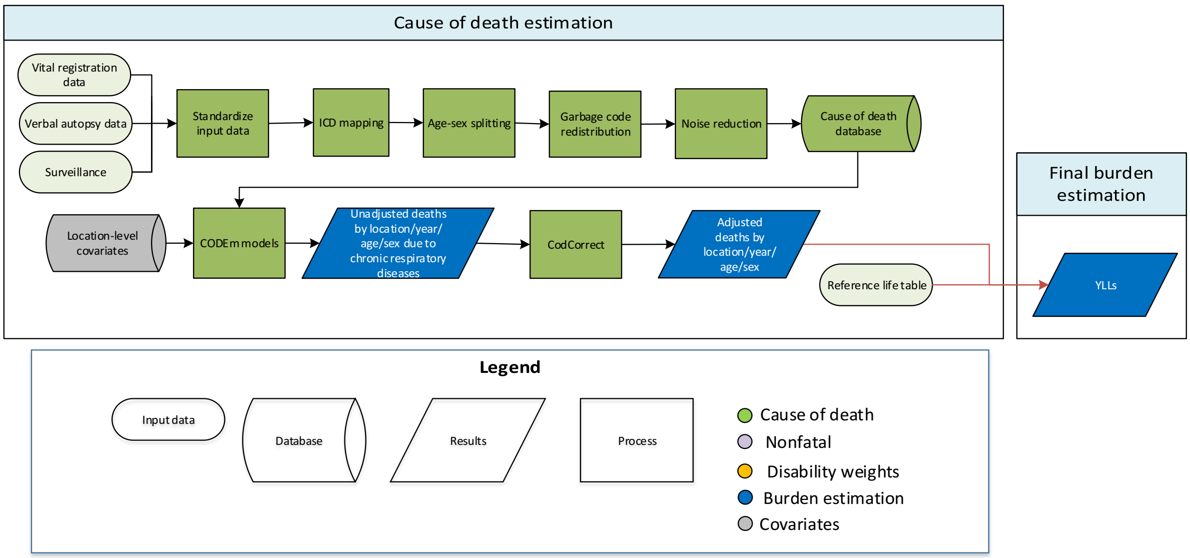

Data

The major data inputs for chronic respiratory disease mortality estimation in India were Sample Registration System (SRS) cause of death data, Medical Certification of Cause of Death (MCCD) data, and other verbal autopsy studies.

SRS is operated by the Office of the Registrar General of India working under the Ministry of Home Affairs, Government of India. Cause of death data from SRS verbal autopsy included 455,460 deaths from the rural and urban populations of every state of India from 2004 to 2013 in which physicians assigned the cause of death based on the information provided in the verbal autopsy interview of a person close to each deceased person.

Using the 2001 census, 7597 geographic units, 4433 (58·4%) of which were rural, were sampled for the 2004–13 SRS to represent the population of each state and union territory of India, ultimately with a sample of 6·7 million people that was equivalent to 0·7% of India's population. The SRS cause of death data for 2004–06, 2007–09, and 2010–13 were provided for each state and union territory by the Office of the Registrar General of India for use in the state-level disease burden estimation. We used 2005, 2008, and 2012 as midpoint years for these three time periods. The inclusion of SRS 2004–13 data in this analysis offers a comprehensive picture of causes of death in India. In the absence of a fully functional vital registration system, verbal autopsy can provide reasonable population level cause of death distribution (Lancet 2017; 390: 2437–2460).

The MCCD system under the Office of the Registrar General of India has data mostly for the urban parts of the states and union territories beginning in 1980. MCCD covered only 22% of the deaths in India in 2015, with the coverage less than 20% in 15 states, 20–50% in ten states and union territories, and more than 50% in some states and union territories. Deaths reported in this data source are medically certified and are considered vital registration data.

In India, cause of death data and verbal autopsy instruments do not classify cause of death by sub-causes of chronic respiratory diseases. These estimates are computed from the overall chronic respiratory disease deaths. Vital registration data from other relevant geographies and covariates were used to estimate mortality levels for the specific chronic respiratory diseases. When data are scarce for a disease or risk factor, GBD uses covariates and techniques that borrow strength from proximity and over time to arrive at the best possible estimates. These were then scaled to sum to the rates from the all chronic respiratory disease model.

Modelling strategy

Mortality estimates for chronic respiratory diseases were generated using the cause of death ensemble modelling (CODEm). CODEm is the framework used to model most cause-specific death rates in the GBD. It relies on four key components. First, all available data are identified and gathered to be used in the modelling process. Though the data may vary in quality, they all contain some signal of the true epidemiological process. Second, a diverse set of plausible models are developed to capture well-documented associations in the estimates. Using a wide variety of individual models to create an ensemble predictive model has been shown to outperform techniques using only a single model both in cause of death estimation and in more general prediction applications. Third, the out-of-sample predictive validity is assessed for all individual models, which are then ranked for use in the ensemble modelling stage. Finally, differently weighted combinations of individual models are evaluated to select the ensemble model with the highest out-of-sample predictive validity.

For some causes, separate models were run for different age ranges when there was reason to believe that the relation between covariates and death rates might be different in different age ranges, for example, in children compared with adults. Separate models are developed for countries with extensive, complete, and representative VR for every cause such that uncertainty can better reflect the more complete vital registration in these locations.

As many factors covary with a particular cause of death, a large range of plausible statistical models are developed for each cause. For the CODEm framework, four families of statistical models are developed using covariates. These are mixed effects linear models of the natural log of the death rate, mixed effects linear models of the logit of the cause fraction, spatiotemporal Gaussian process regression (ST-GPR) models of the log of the death rate, and ST-GPR of the logit of the cause fraction. All plausible relationships between covariates and relevant cause are identified, and all possible permutations of selected covariates are tested in linear models where the logit cause fraction or log death rate is the response variable. Because we test all permutations of covariates, multicollinearity between covariates may produce implausible signs on coefficients or unstable coefficients. All models where the sign on the coefficient is in the direction expected based on the literature and where the coefficient is statistically significant at $p < 0.05$ are retained. We run covariate selection for both cause fractions and death rates and then create both mixed effects only and ST models for each set of covariates.

The performance of all component models and ensembles is evaluated using out-of-sample predictive validity tests. Thirty percent of the data are excluded from the initial model fits, and half of that (15% of total) is used to evaluate and rank component models and then build ensembles. Data are held out from the analysis using the pattern of missingness for each cause in the cause of death database. Out-of-sample predictive validity testing is repeated until stable model results have been obtained. The out-of-sample performance tests include the root mean squared error of the log of the cause-specific death rate, the direction of the trend in the prediction

compared to the data, and the validity of the 95% UI. For every model, we show the in-sample root mean squared error of the log death rates (RMSE) and the out-of-sample performance in the 15% of data not used in the model building process.

After component models are ranked on their out-of-sample predictive validity they are weighted based on their ranking and each component model contributes a portion to the final estimate. How much each submodel contributes is a function of its relative ranking as well as the value of psi chosen, which dictates that distribution of rankings.

Using the second half of the holdout data (15% of total), the differently weighted ensembles and different values of psi are tested using the same predictive validity metrics as the component models. For every model, we show the in-sample RMSE and the out-of-sample performance in the 15% of data not used in the model building process. The ensemble with the best average trend and RMSE is chosen as the final ensemble weighting scheme.

After a model weighting scheme has been chosen, each model contributes a number of draws proportional to its weight such that 1,000 draws are created. The mean of the draws is used as the final estimate for the CODEm process and 95% UI are created from the 0.025 and 0.975 quantiles of the draws. The final assessment of ensemble model performance is the validity of the UIs; ideally, the 95% UI for a model would capture 95% of the data out-of-sample. Higher coverage suggests that UIs are too large and lower than 95% suggest UIs are too narrow.

Chronic respiratory diseases served as the parent cause to chronic obstructive pulmonary disease, pneumoconiosis (including silicosis, asbestosis, coal worker's pneumoconiosis, other pneumoconiosis), asthma, interstitial lung disease and pulmonary sarcoidosis, and other chronic respiratory diseases. Functionally, this means the death estimates for chronic respiratory diseases serve as a "parent" envelope into which the "child" causes are squeezed by the CodCorrect algorithm. This approach allows us to use a broader range of data – specifically verbal autopsy data – which cannot be accurately mapped to specific respiratory diseases. Separate models were conducted for male and female mortality, and the age range for both models was 0 to 95+ years.

CODEm models estimate the individual cause-level mortality without taking into account the all-cause mortality. GBD uses the CodCorrect algorithm to ensure that all individual causes add up to the all-cause mortality. After generating underlying cause of death estimates and accompanying uncertainty, this algorithm combines these models into estimates that are consistent with the levels of all-cause mortality estimated for each age-sex-year-location group. Using 1000 draws from the posterior distribution of each cause and 1000 draws from the posterior distribution of the estimation of all-cause mortality, CoDCorrect rescales the sum of cause-specific estimates to equal the draws from the all cause distribution. Further details of CodCorrect algorithm can be found in the appendix to the GBD 2016 cause of death capstone paper (Lancet 2017; 390: 1151–210).

The covariates included in the model were:

Level	Covariate	Direction
1	Log-transformed SEV scalar: chronic respiratory diseases	+
	Cumulative cigarettes (10 years)	+
	Cumulative cigarettes (5 years)	+
	Health care quality and access index	-
2	Smoking prevalence	+
	Indoor air pollution (all cooking fuels)	+
	Outdoor air pollution ($PM_{2.5}$)	+
	Population above 1500m elevation (proportion)	+
3	Log LDI (I$ per capita)	-
	Education (years per capita)	-
	Socio-demographic Index	-
	Population between 500 and 1,500m elevation (proportion)	+

	Population density over 1,000 people/square meter (proportion)	+

The covariates used for the estimation of deaths due to COPD were:

Level	Covariate	Direction
1	Log-transformed SEV scalar: COPD	+
	Cumulative cigarettes (10 years)	+
	Cumulative cigarettes (5 years)	+
	Elevation over 1,500m (proportion)	+
2	Smoking prevalence	+
	Indoor air pollution (all cooking fuels)	+
	Outdoor air pollution ($PM_{2.5}$)	+
	Health care access and quality index	-
3	Socio-demographic Index	-
	Log LDI (I$ per capita)	-
	Education (years per capita)	-

The covariates used for the estimation of deaths due to asthma were:

Level	Covariate	Direction
1	Log-transformed SEV scalar: asthma	+
	Cumulative cigarettes (10 years)	+
	Cumulative cigarettes (5 years)	+
	Health care access and quality index	-
2	Smoking prevalence	+
	Indoor air pollution (all cooking fuels)	+
	Outdoor air pollution ($PM_{2.5}$)	+
3	Log LDI (I$ per capita)	-
	Education (years per capita)	-
	Socio-demographic Index	-

In the above tables, level indicates the proximity of the covariate in the causal chain and a judgment on the strength of the causal relationship (level 1: strong plausible causal relationship between a risk and outcome; level 2: associated with outcome, less strong evidence; level 3 more distal predictors such as education or LDI. Direction: + if strong belief for a positive relationship; - if strong belief for a negative relationship; 0 for covariates that could have a relationship in either direction. Lagged distributed income or LDI reflects gross domestic product (GDP) data with a lag built as it takes some years for changes in GDP to impact on health. The SEV scalar for a disease is a compound measure of exposure to all risks estimated in GBD to have an impact on that disease, weighted by the relative risk for each exposure. Socio-demographic Index (SDI) is a compound measure of LDI, average years of schooling in population over 15 and total fertility rate.

F. Estimation of major risk factors for chronic respiratory diseases

The approach used in GBD 2016 for comparative risk assessment to estimate population attributable fractions for risk factors is shown in the following flowchart.

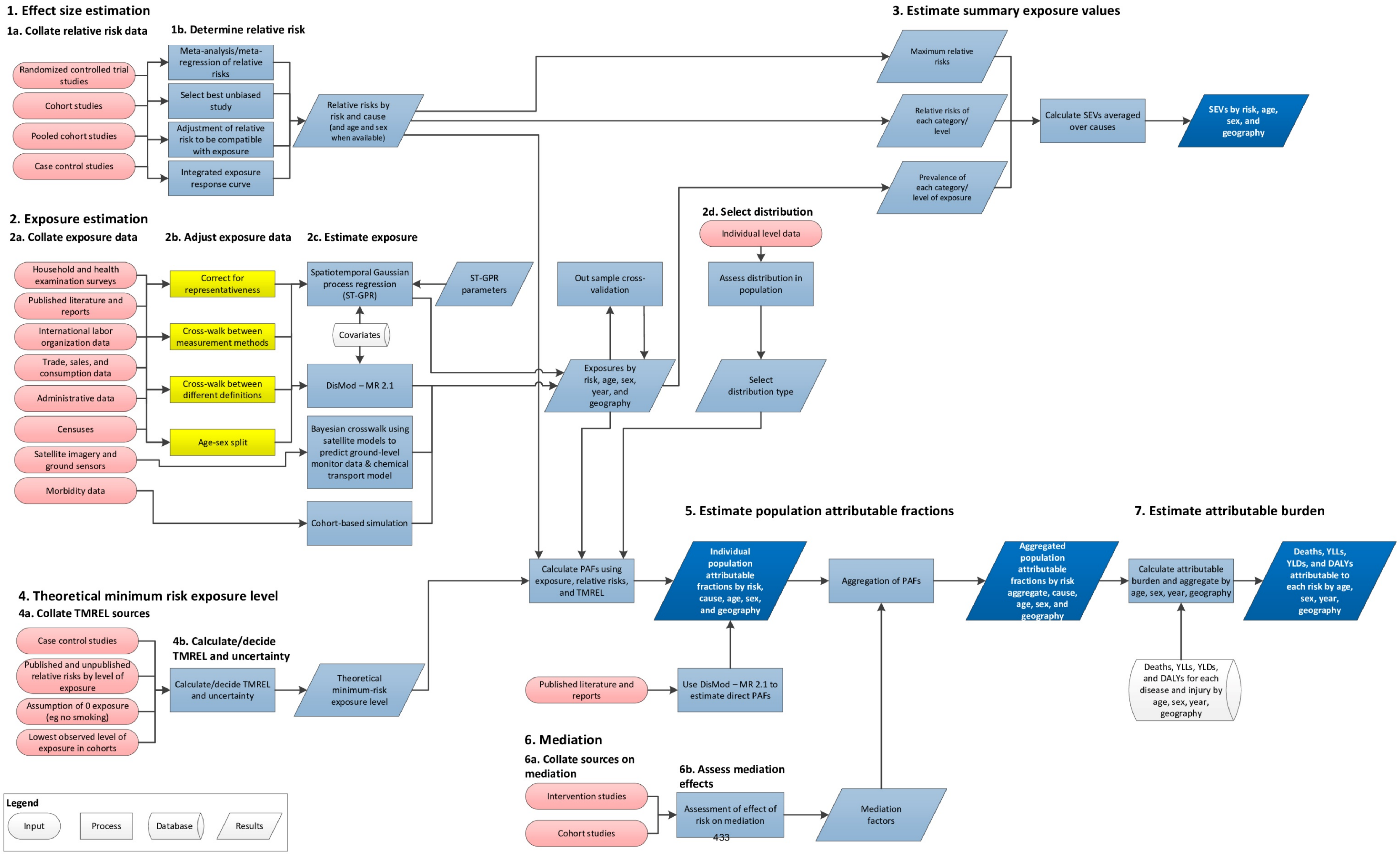

GBD is Global Burden of Disease. SEV is summary exposure value. TMREL is theoretical minimum-risk exposure level. PAF is population attributable fraction. YLL is years of life lost. YLD is years lived with disability. DALY is disability-adjusted life-year. Ovals represent data inputs, rectangular boxes represent analytical steps, cylinders represent databases, and parallelograms represent intermediate and final results.

We describe details of three major risk factors related to chronic respiratory diseases that are elaborated in this paper, i.e. ambient particulate matter pollution, household air pollution**,** and smoking. Description of other risk factors is available in the GBD 2016 risk factors capstone paper (Lancet 2017; 390: 1345–422).

F.1. Ambient particulate matter pollution

Exposure to ambient air pollution for this GBD analysis was defined as the population-weighted annual average mass concentration of particles with an aerodynamic diameter less than 2.5 micrometers ($PM_{2.5}$) in a cubic meter of air at a spatial resolution of approximately 11 x 11 km. This measurement was reported in $\mu g/m^3$.

For the purpose of attributing disease burden to ambient air pollution, the theoretical minimum-risk exposure level for ambient air pollution was defined as population-weighted mean between 2.4 and 5.9 $\mu g/m^3$, bounded by the minimum and fifth percentiles of exposure distributions from outdoor air pollution cohort studies. The uniform distribution represents the uncertainty regarding adverse effects of low-level exposure.

The steps in the estimation of disease burden attributable to ambient air pollution are shown in the following flowchart:

Ambient PM2.5

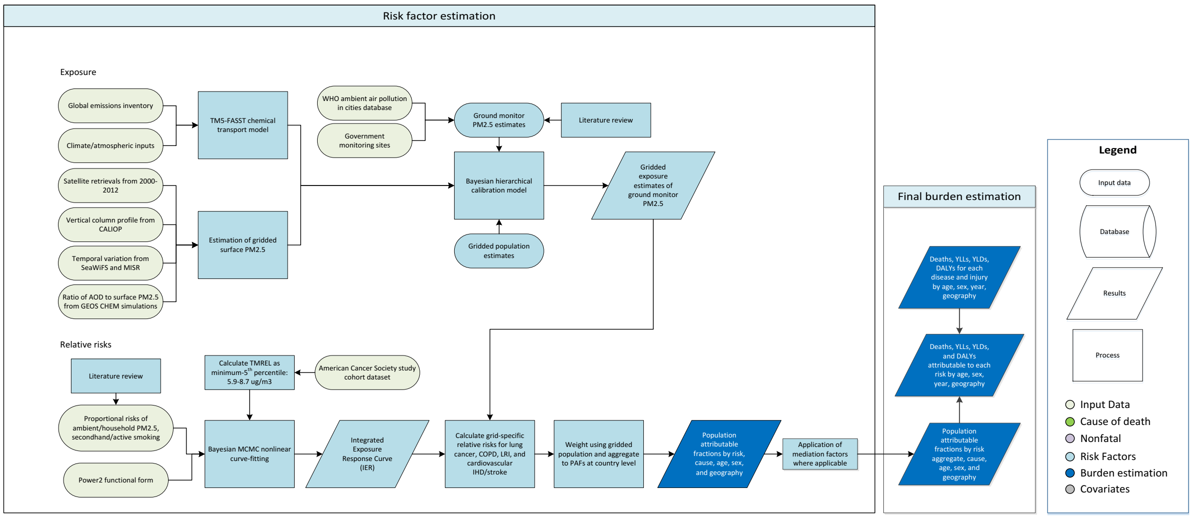

Data

The estimates of ambient $PM_{2.5}$ exposures in India were based on multiple satellite-based aerosol optical depth data combined with a chemical transport model, and calibration of these with $PM_{2.5}$ data from the ground-level monitoring stations.

$PM_{2.5}$ ground measurements: Monitor-specific measurements (rather than city averages as reported in the WHO Air Pollution in Cities database) were used, resulting in measurements of concentrations of PM_{10} and $PM_{2.5}$ from over 6,000 ground monitors from 117 countries. For locations measuring only PM_{10}, $PM_{2.5}$ measurements were estimated from PM_{10}. This was performed using a locally derived conversion factor ($PM_{2.5}/PM_{10}$ ratio, for stations where measurements are available for the same year) that was estimated using population-weighted averages of location-specific conversion factors for the country or state. If country-level conversion factors were not available, the average of country-level conversion factors within a region were used. Additional information related to the ground measurements was also included where available, including monitor geo coordinates and monitor site type.

Satellite-based estimates: These estimates were available at 0.1×0.1^o resolution (~11 x 11 km resolution at the equator) and combine aerosol optical depth retrievals from multiple satellites with the GEOS Chem chemical transport model and land use information.

Population data: A comprehensive set of population data on a high-resolution grid was obtained from the Gridded Population of the World (GPW) database. These data are provided on a 0.0417×0.0417^o resolution.

Aggregation to each 0.1×0.1^o grid cell comprised of summing the central 3 × 3 population cells. As this resulted in a resolution higher than necessary, it was repeated four times, each offset by one cell in a North, South, East and West direction. The average of the resulting five quantities was used as the estimated population for each grid cell.

Chemical transport model simulations: Estimates of the sum of particulate sulfate, nitrate, ammonium and organic carbon and the compositional concentrations of mineral dust simulated using the GEOS Chem chemical transport model, and a measure combining elevation and the distance to the nearest urban land surface were available for 2000 to 2015 for each 0.1×0.1^o grid cell.

Modelling strategy

Global annual mean exposure to $PM_{2.5}$ was estimated in 5-year intervals from 1990 to 2015, at 0·1 × 0·1° (~11 km × 11 km at the equator) resolution using estimates from satellites combined with a chemical transport model, surface measurements, and geographical data. We aggregated gridded exposure concentrations to national-level population-weighted means using the corresponding grid cell population value. National-level population-weighted mean concentrations and the 95% uncertainty interval (95% UI) around this mean were estimated by sampling 1000 draws of each grid cell value and its uncertainty distribution. We used a chemical transport model to calculate a running 3-month mean.

The Data Integration Model for Air Quality (DIMAQ) was used for ambient air pollution modelling. The coefficients in the calibration model were estimated for each country or state. Where data were insufficient within a country or state, information can be `borrowed' from a higher aggregation (region) and if enough information is still not available from an even higher level (super region). Individual country level estimates were therefore based on a combination of information from the country, its region and super-region. This was implemented within a Bayesian Hierarchical Modelling (BHM) framework. BHMs provide an extremely useful and flexible framework in which to model complex relationships and dependencies in data. Uncertainty can also be propagated through the model allowing uncertainty arising from different components, both data sources and models, to be incorporated within estimates of uncertainty associated with the final estimates. The results of the modelling comprise a posterior distribution for each grid cell, rather than just a single point estimate, allowing a variety of summaries to be calculated. The primary outputs here are the median and 95% credible intervals for each grid cell.

Due to both the complexity of the models and the size of the data, notably the number of spatial predictions that are required, recently developed techniques that perform ‘approximate’ Bayesian inference based on Integrated Nested Laplace Approximations (INLA) were used. Computation was performed using the R interface to the INLA computational engine (R-INLA). Fitting the models and performing predictions for each of the ca. 1.4 million grid cells required the use of a high performance computing cluster (HPC) making use of high memory nodes.

Model development and comparison was performed using within- and out-of-sample assessment. In the evaluation, cross validation was performed using 25 combinations of training (80%) and validation (20%) datasets. Validation sets were obtained by taking a stratified random sample, using sampling probabilities based on the cross-tabulation of $PM_{2.5}$ categories (0-24.9, 25-49.9, 50-74.9, 75-99.9, 100+ $\mu g/m^3$) and super-regions, resulting in them having the same distribution of $PM_{2.5}$ concentrations and super-regions as the overall set of sites. The following metrics were calculated for each training/evaluation set combination: for model fit - R^2 and deviance information criteria (DIC, a measure of model fit for Bayesian models); for predictive accuracy - root mean squared error (RMSE) and population weighted root mean squared error (PwRMSE).

All modelling was performed on the log-scale. The choice of which variables were included in the model was made based on their contribution to model fit and predictive ability. The following is a list of variables and model structures that were considered in developing the model:

**Variable**	**Model structure**
Continuous explanatory variables	(SAT) Estimate of $PM_{2.5}$ (in µgm-3) for 2014 from satellite remote sensing on the log scale.
	(CTM) Estimate of $PM_{2.5}$ (in µgm-3) for 2010 from the TM5 chemical transport model on the log-scale.
	(POP) Estimate of population for 2014 on the log-scale.
	(SNAOC) Estimate of the sum of sulfate, nitrate, ammonium and organic carbon simulated using the GEOS Chem chemical transport model.

	(DST) Estimate of compositional concentrations of mineral dust simulated using the GEOS Chem chemical transport model.
	(EDxDU) The log of the elevation difference between the elevation at the ground measurement location and the mean elevation within the GEOS Chem simulation grid cell multiplied by the inverse distance to the nearest urban land surface.
Discrete explanatory variables	(LOC) Binary variable indicating whether exact location of ground measurement is known.
	(TYPE) Binary variable indicating whether exact type of ground monitor is known.
	(CONV) Binary variable indicating whether ground measurement is $PM_{2.5}$ or converted from PM_{10}.
Random Effects	Grid cell random effects on the intercept to allow for multiple ground monitors in a grid cell.
	Country-region-super-region hierarchical random effects for the intercept.
	Country-region-super-region hierarchical random effects for the coefficient associated with SAT.
	Country-region-super-region hierarchical random effects for the coefficient associated with the difference between estimates from CTM and SAT.
	Country-region-super-region hierarchical random effects for the coefficient associated with POP.
	Country level random effects for population uses a neighbourhood structure allowing specific borrowing of information from neighbouring countries.
	Within a region, country level effects of SAT and the difference between SAT AND CTM are assumed to be independent and identically distributed.
	Within a super-region, region level random effects are assumed to be independent and identically distributed.
	Super-region random effects are assumed to be independent and identically distributed.
Interactions	Interactions between the binary variables and the effects of SAT and CTM.

The final model contained the following variables: SAT, POP, SNAOC, DST, EDxDU, LOC, TYPE, and CONV, together with interactions between SAT and each of LOC, TYPE and CONV. The model structure contained grid cell random effects on the intercept to allow for multiple ground monitors in a grid cell, country-region-super-region hierarchical random effects for intercepts and SAT and country level random effects for population using a neighbourhood structure allowing specific borrowing of information from neighbouring countries together with region-super-region hierarchical random effects for POP.

DIMAQ showed improved predictions of ground measurements in all super regions. Using this model resulted in an improvement in both within-sample fit; with an increase in R^2 to 0.91, and out-of- sample predictive ability; with a global population-weighted RMSE of 12.1 µg/m3.

Satellite estimates, populations and quantities estimated using the GEOS-Chem model were available for 1990, 1995, 2000, 2005, 2010, 2011, 2012, 2013, 2014 and 2015. Population estimates for 2000, 2005, 2010, 2015 and 2020 were available from GPW version 4. For 1990 and 1995 data were extracted from GPW version 3. As with populations for 2015, values for each cell for 2011, 2012, 2012, 2013 and 2014 were obtained by interpolation using natural splines with knots placed at 2000, 2005, 2010, 2015 and 2020.

These were used as inputs to DIMAQ, enabling estimates of exposures to be obtained for each of these years respectively. For 2016, estimates of exposures were obtained from predictions from locally-varying regression models. For each cell a model was fit to the values within that cell over time, with a constraint placed on the rate of change between 2015 and 2016 to avoid unrealistic and/or unjustified extrapolation of trends. Measures of uncertainty were obtained by repeating the procedure for the limits of the 95% intervals, again on a cell-by-cell basis.

We estimated the burden attributable to $PM_{2.5}$ for ischaemic heart disease (IHD), stroke, lung cancer, COPD, and lower respiratory infections (LRI). Evidence linking these diseases with exposure to ambient air pollution was judged to be consistent with a causal relationship on the basis of criteria specified for GBD risk factors. We developed integrated exposure–response functions (IERs) for each cause of death to estimate the relative risk of mortality over the entire global range of ambient annual mean $PM_{2.5}$ concentrations using risk estimates from studies of ambient air pollution, household air pollution, and second-hand smoke exposure and active smoking. IERs assign concentrations of $PM_{2.5}$ to each type of exposure on an equivalent µg/m³ basis assuming that risk is determined by the 24-h $PM_{2.5}$ inhaled dose regardless of the exposure source. An alternative method to estimate exposure to second-hand smoke was used that incorporated estimates of $PM_{2.5}$ attributable to

exposure per cigarette, breathing rate, and number of cigarettes smoked in the country where each study was done.

The IER has the mathematical form:

$$IER(\beta, z) = 1 + \alpha \times (1 - e^{-\beta(z - z_{cf})_+^{\gamma}})$$

where z is the level of $PM_{2.5}$ and z_{cf} is the TMREL, below which no additional risk is assumed, with

$$(z - z_{cf})_+ = (z - z_{cf})$$

if z is greater than z_{cf} and zero otherwise. Here, 1 + α is the maximum risk, β is the ratio of the IER at low to high concentrations, and γ is the power of $PM_{2.5}$ concentration. Epidemiological evidence suggests that the relative risks for IHD and stroke decline with age. We modified the particulate matter source-specific relative risk for both IHD and stroke mortality and applied this age modification to the relative risks, fitting the IER model for each age group separately. Observed relative risks were related to the IER within a Bayesian framework using the STAN fitting algorithm. Given the true values of the four parameters (α, β, γ, z_{cf}), we assumed that the logarithm of each study's observed relative risk was normally distributed, with mean defined by the IER and variance given by the square of the observed SE of the study-specific log-relative risk estimate plus an additional variance term for each of the four sources on $PM_{2.5}$ exposure (outdoor air pollution, second-hand smoke, household air pollution, and active smoking).

We calculated 1000 predicted values of the IER for each $PM_{2.5}$ concentration based on the posterior distributions of (α, β, γ) and the prespecified uniform distribution of TMREL to characterise uncertainty in the estimates of the IER. The mean of the 1000 IER predictions at each concentration was used as the central estimate, with uncertainty defined by 95% UIs.

F.2. Household air pollution

Exposure to household air pollution from solid fuels (HAP) for this GBD analysis was defined as the proportion of households using solid cooking fuels. The definition of solid fuel in this analysis includes coal, wood, charcoal, dung, and agricultural residues.

The steps in the estimation of disease burden attributable to household air pollution are shown in the following flowchart:

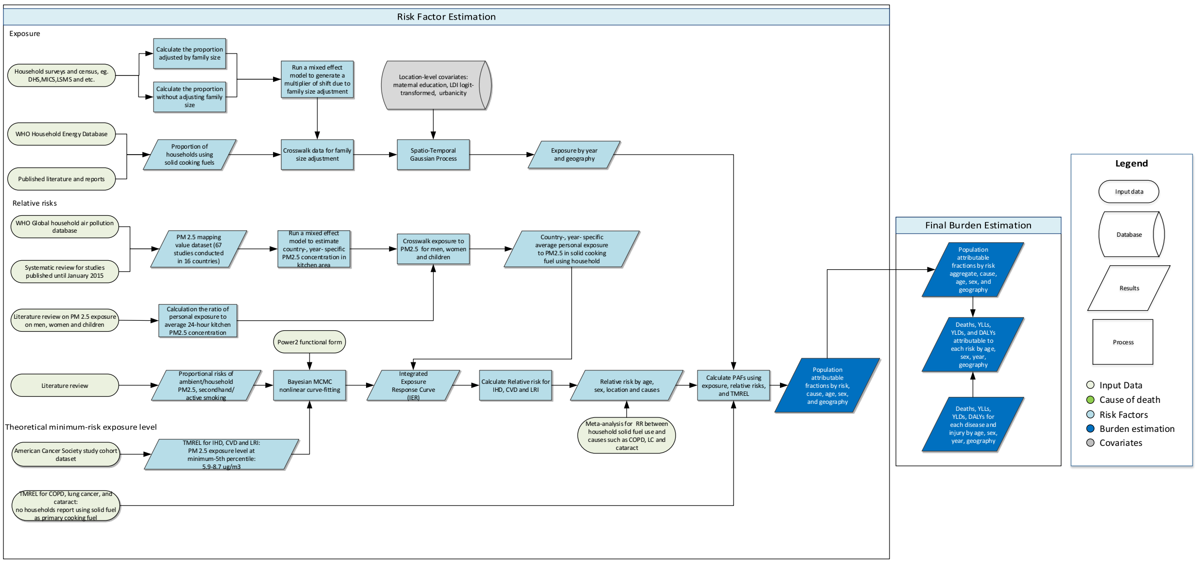

Data

The major data sources on household air pollution from solid fuel use in India include national health surveys such as the National Family Health Survey and the District Level Household Survey, nationwide surveys of the National Sample Survey Organisation, and the Census of India, as well as other published and unpublished epidemiological studies.

Data were extracted from the standard multi-country survey series such as Demographic and Health Surveys (DHS), Living Standards Measurement Surveys (LSMS), Multiple Indicator Cluster Surveys (MICS), and World Health Surveys (WHS), as well as country-specific survey series. To fill the gaps of data in surveys and censuses, we also downloaded and updated HAP estimates from WHO Energy Database and extracted from literature through systematic review. Each nationally or sub-nationally representative data point provided an estimate for the percentage of households using solid cooking fuels. Estimates for the usage of solid fuels for non-cooking purpose were excluded, i.e. primary fuels for lighting.

Modelling strategy

Household air pollution was modelled at household level using a three-step modelling strategy that uses linear regression, spatiotemporal regression and Gaussian Process Regression (GPR). The first step is a mixed-effect linear regression of logit-transformed proportion of households using solid cooking fuels. The linear model contains maternal education, proportion of population living in urban areas, and lagged-distributed income as covariates and has nested random effect by GBD region, and GBD super region respectively.

A variety of combinations of socioeconomic and environmental covariates in different transformation format were tested by running mixed-effect models with exposure data. The final list of covariates included in the exposure model are maternal education, proportion of population living in urban area, and lagged-distributed income since they proved to be the strongest predictors.

The disease-outcomes paired with household air pollution include lower respiratory infections (LRI), stroke, Ischemic Heart Disease (IHD), COPD, lung cancer and cataract. The relative risks of all outcomes, with the exception of cataracts, were generated by using the integrated exposure-response functions (IER), as previously described in the section on ambient air pollution modelling. This is done by first estimating the crosswalk values that map household use of solid fuel to $PM_{2.5}$ exposure because the IER curve measures exposure using $PM_{2.5}$. The average $PM_{2.5}$ exposures from solid fuel use for different household members were derived from studies measuring 24-hour kitchen and living area $PM_{2.5}$ concentrations in households, and estimating this for men, women and children. For outcomes that utilise evidence based on the Integrated Exposure Response, the TMREL was defined as uniform distribution of exposure to $PM_{2.5}$ from solid fuel use between 2.4 and 5.9 ug/m^3. The relative risks for cataracts were extracted from a meta-analysis paper (*Annu Rev Public Health* 2014; 35: 185–206).

F.3. Smoking

For the purpose of attributing disease burden to smoking, the TMREL was all individuals who were lifelong non-smokers, above which there could be adverse health effects.

The steps in the estimation of disease burden attributable to smoking are shown in the following flowchart:

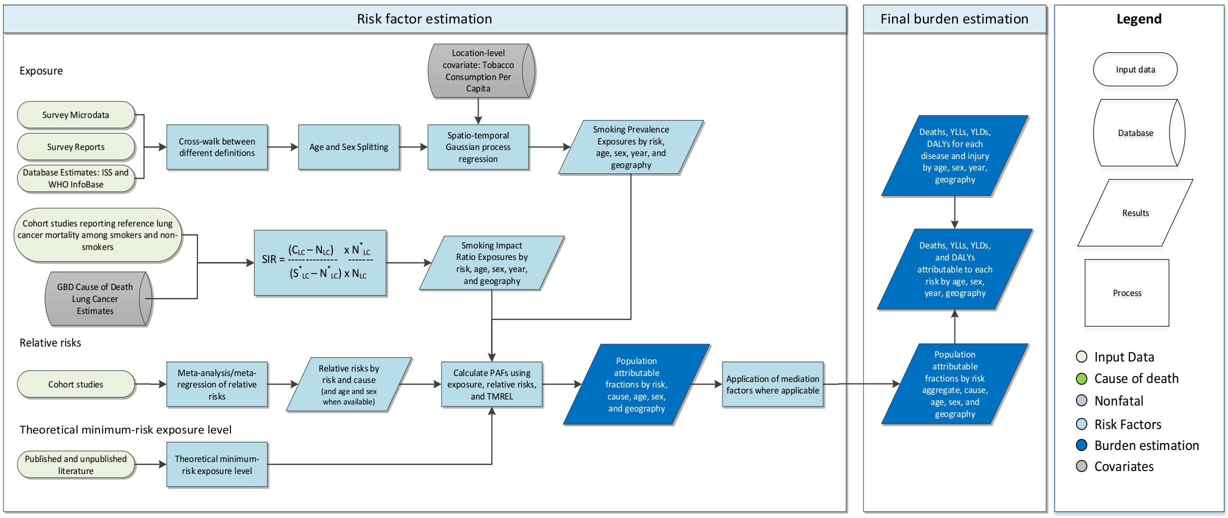

Data

We included representative survey data sources that captured information on primary tobacco use among individuals over age 10. We included only self-reported smoking data and excluded data from questions asking about others' smoking behaviours. In addition to the primary data sources, we used secondary database estimates from the WHO InfoBase Database and International Smoking Statistics Database for sources for which primary data are unavailable.

We extracted primary data from individual-level microdata and survey report tabulations. We extracted data on current smoked tobacco use reported as any combination of frequency of use (daily, occasional, and current, which includes both daily and occasional smokers), type of smoked tobacco used (all smoked tobacco, cigarettes, hookah, and other smoked tobacco products such as cigars or pipes), and whether the data included only current smokers, only former smokers, or both current and former smokers, resulting in 36 possible combinations.

For microdata, we extracted relevant demographic information, including age, sex, location, and year, as well as survey metadata, including survey weights, primary sampling units, and strata. This information allowed us to tabulate individual-level data in the standard GBD five-year age-sex groups and produce accurate estimates of uncertainty. For survey report tabulations, we extracted data at the most granular age-sex group provided.

Crosswalking: Our case-definition for smoking prevalence is current daily use of any smoked tobacco products. All other data points were adjusted to be consistent with this definition. Some sources contained information on more than one indicator and these sources were used to develop the adjustment coefficient to transform that alternative definitions to the GBD standard case-definition of daily use of smoked tobacco. The adjustment coefficient was the beta value derived from the following model:

$$p_{daily-smoked,k} = \beta p_{i,k} + \epsilon_k$$

where $p_{daily-smoked,k}$ is the prevalence of daily smoking reported in survey k and $p_{i,k}$ is the prevalence of an alternative frequency-type combination i also reported in survey k. Models with adjusted R-squared values > 0.8 were used in order of their R-squared value.

We propagated uncertainty at the survey (k*) level* from the crosswalk using the following equation:

$$PE_k = \sigma_\epsilon^2 + X_k^2 var(\hat{\beta})$$

where PE_k is the crosswalk prediction error that is added to the sampling variance of the data point, σ_ϵ^2 is the variance of the error, X_k^2 is the squared value of the data being adjusted, and $var(\hat{\beta})$ is the variance of the adjustment coefficient.

We split data reported in broader age groups than the GBD 5-year age groups or as both sexes combined using a standard approach.

Modelling strategy

We used ST-GPR to model smoking prevalence given the abundance of age and sex-specific data. Briefly, the mean function input to GPR is a complete time series of estimates generated from a mixed effects hierarchical linear model plus weighted residuals smoothed across time, space, and age. The linear model formula, fit separately by sex using restricted maximum likelihood in R, is:

$$\text{logit}(p_{g,a,t}) = \beta_0 + \beta_1 CPC_{g,t} + \sum_{k=2}^{19} \beta_k I_{A[a]} + \alpha_s + \alpha_r + \alpha_g + \epsilon_{g,a,t}$$

where $CPC_{c,t}$ is the tobacco consumption covariate, by geography g and time t, described above, $I_{A[a]}$ is a dummy variable indicating specific age group A that the prevalence point $p_{g,a,t}$ captures, and α_s, α_r, and α_g are super region, region, and geography random intercepts, respectively. Random effects were used in model fitting but were not used in prediction.

We used out-of-sample cross validation for hyperparameter selection for the space (zeta), age (omega), and time (lambda) weights used in spatiotemporal smoothing along with the scale used in Gaussian process regression (details on the effects of different parameters have been previously published). We used a space weight of 0.95 in data-dense countries (at least five years covered in a geography-age-sex group) and space weight of 0.7 in data-sparse countries. The other parameters were consistent across data-density levels: age weight = 1, time weight = 1, and scale = 10.

Relative risk estimates were derived from prospective cohort studies. Relative risk estimates and uncertainty for all outcomes associated with smoking were included in analysis, by age and sex as applicable.

G. Uncertainty intervals

Point estimates for each quantity of interest were derived from the mean of the draws, while 95% uncertainty intervals (UIs) were derived from the 2.5th and 97.5th percentiles of the 1000 draw level values. Uncertainty in the estimation is attributable to sample size variability within data sources, different availability of data by age, sex, year, or location, and cause specific model specifications. We determined UIs for components of cause-specific estimation based on 1000 draws from the posterior distribution of cause specific mortality by age, sex, and location for each year included in the GBD 2016 analysis. Similarly, for non-fatal estimates if there was a change in disease estimates between locations or over time that was in the same direction in more than 950 of the 1000 samples we report it as significant. With this approach, uncertainty could be quantified and propagated into the final quantities of interest.

2. GBD 2016 India data inputs for chronic respiratory diseases mortality, morbidity, risk factors, and covariates

Agrawal S. Effect of indoor air pollution from biomass and solid fuel combustion on prevalence of self-reported asthma among adult men and women in India: findings from a nationwide large-scale cross-sectional survey. J Asthma. 2012; 49(4): 355-65.
Ali MK, Bhaskarapillai B, Shivashankar R, Mohan D, Fatmi ZA, Pradeepa R, Masood Kadir M, Mohan V, Tandon N, Narayan KM, Prabhakaran D, CARRS investigators. Socioeconomic status and cardiovascular risk in urban South Asia: The CARRS Study. Eur J Prev Cardiol. 2016; 23(4): 408-19.
Asthma Bhawan Jaipur, International Study of Asthma and Allergies in Childhood (ISAAC) Steering Committee, ISAAC Phase Three Study Group, ISAAC International Data Centre Auckland. India-ISAAC Phase-3 data 2001-2003 [Data shared for this analysis].
Bang AT, Bang RA, Baitule SB, Reddy MH, Deshmukh MD. Effect of home-based neonatal care and management of sepsis on neonatal mortality: field trial in rural India. Lancet. 1999; 354(9194): 1955-61.
Baruah J, Kusre G, Bora R. Pattern of Gross Congenital Malformations in a Tertiary Referral Hospital in Northeast India. Indian J Pediatr. 2015; 82(10): 917–22.
Behl RK, Kashyap S, Sarkar M. Prevalence of bronchial asthma in school children of 6-13 years of age in Shimla city. Indian J Chest Dis Allied Sci. 2010; 52(3): 145-8.
Bhandari N, Mazumder S, Taneja S, Sommerfelt H, Strand TA. Effect of implementation of Integrated Management of Neonatal and Childhood Illness (IMNCI) programme on neonatal and infant mortality: cluster randomised controlled trial. BMJ. 2012; 344: e1634.
Bharaj P, Sullender WM, Kabra SK, Mani K, Cherian J, Tyagi V, Chahar HS, Kaushik S, Dar L, Broor S. Respiratory viral infections detected by multiplex PCR among pediatric patients with lower respiratory tract infections seen at an urban hospital in Delhi from 2005 to 2007. Virol J. 2009; 6: 89.
Blencowe H, Cousens S, Jassir FB, Say L, Chou D, Mathers C, Hogan D, Shiekh S, Qureshi ZU, You D, Lawn JE; Lancet Stillbirth Epidemiology Investigator Group. National, regional, and worldwide estimates of stillbirth rates in 2015, with trends from 2000: a systematic analysis. Lancet Glob Health. 2016; 4(2): e98-108.
BP. BP Statistical Review of World Energy 2016. London, United Kingdom: BP; 2016.
British Geological Survey, Natural Environment Research Council. World Mineral Production 2007-2011. Nottingham, United Kingdom: British Geological Survey; 2013.
Buist AS, McBurnie MA, Vollmer WM, Gillespie S, Burney P, Mannino DM, Menezes AM, Sullivan SD, Lee TA, Weiss KB, Jensen RL, Marks GB, Gulsvik A, Nizankowska-Mogilnicka E, BOLD Collaborative Research Group. International variation in the prevalence of COPD (the BOLD Study): a population-based prevalence study. Lancet. 2007; 9589(9589): 741-50.
Central Board of Secondary Education, Department of School Education and Literacy, Ministry of Human Resource Development, Government of India, Centers for Disease Control and Prevention, World Health Organization (WHO). India Global School-Based Student Health Survey 2007. Geneva, Switzerland: WHO; 2007.
Central Pollution Control Board (CPCB), Ministry of Environment, Forest and Climate Change, Government of India. India - National Air Quality Monitoring Programme Data 2012. New Delhi, India: CPCB.
Central Pollution Control Board (CPCB), Ministry of Environment, Forest and Climate Change, Government of India. India - National Air Quality Monitoring Programme Data 2013. New Delhi, India: CPCB.
Chest Research Foundation Pune, KEM Hospital Research Centre Pune, Population Health and Occupational Disease, National Heart & Lung Institute, Imperial College London. India - Pune Burden of Obstructive Lung Disease Initiative Survey (BOLD) 2008-2009. [Data shared for this analysis]
Cho SH, Lin HC, Ghoshal AG, Muttalif BA, Razak A, Thanaviratananich S, et al. Respiratory disease in the Asia-Pacific region: cough as a key symptom. In: Allergy Asthma Proc. OceanSide Publications, Inc; 2016; 37(2): 131–40.
Chowgule RV, Shetye VM, Parmar JR, Bhosale AM, Khandagale MR, Phalnitkar SV, Gupta PC. Prevalence of respiratory symptoms, bronchial hyperreactivity, and asthma in a megacity. Results of the European community respiratory health survey in Mumbai (Bombay). Am J Respir Crit Care Med. 1998; 158(2): 547-54.
Christian Medical College - Vellore. India Prevalence of Risk Factors for Non-Communicable Diseases in Rural and Urban Tamil Nadu 2010-2012. [Data shared for this analysis]
Coal Controller's Organization, Ministry of Coal, Government of India. India Provisional Coal Statistics 2014-2015. Kolkata, India: Coal Controller's Organization, Ministry of Coal; 2015.
Department of Pulmonary Medicine, Postgraduate Institute of Medical Education & Research. India Study on Epidemiology of Asthma, Respiratory Symptoms and Chronic Bronchitis (INSEARCH) 2004-2005. [Data shared for this analysis]
Department of Pulmonary Medicine, Postgraduate Institute of Medical Education & Research. India Study on Epidemiology of Asthma, Respiratory Symptoms and Chronic Bronchitis (INSEARCH) 2007-2009. [Data shared for this analysis]
Department of Pulmonary Medicine, Postgraduate Institute of Medical Education & Research. India Study on Epidemiology of Asthma, Respiratory Symptoms and Chronic Bronchitis 2004-2005. [Data shared for this analysis]
Department of Women and Child Development, Ministry of Human Resource Development, Government of India, United Nations Children's Fund (UNICEF) - India Country Office. India Summary Report on the Multiple Indicator Cluster Survey 2000. New Delhi, India: UNICEF India Country Office; 2000.

Desai, Sonalde, Reeve Vanneman, National Council of Applied Economic Research, University of Michigan. India Human Development Survey 2005. Ann Arbor, Michigan: Inter-University Consortium for Political and Social Research.

Dhabadi BB, Athavale A, Meundi A, Rekha R, Suruliraman M, Shreeranga A, Gururaj S. Prevalence of asthma and associated factors among schoolchildren in rural South India. Int J Tuberc Lung Dis. 2012; 16(1): 120-5.

Directorate of Economics & Statistics and Office of Chief Registrar (Births & Deaths), Government of National Capital Territory of Delhi. Report on Medical Certification of Cause of Deaths in Delhi-2011. New Delhi, India: Directorate of Economics & Statistics and Office of Chief Registrar (Births & Deaths); 2012.

Directorate of Economics & Statistics and Office of Chief Registrar (Births & Deaths), Government of National Capital Territory of Delhi. Report on Medical Certification of Cause of Deaths in Delhi-2012. New Delhi, India: Directorate of Economics & Statistics and Office of Chief Registrar (Births & Deaths); 2014.

Directorate of Economics & Statistics and Office of Chief Registrar (Births & Deaths), Government of National Capital Territory of Delhi. Report on Medical Certification of Cause of Deaths in Delhi-2013. New Delhi, India: Directorate of Economics & Statistics and Office of Chief Registrar (Births & Deaths); 2014.

Ellwood P, International Study of Asthma and Allergies in Childhood (ISAAC) Steering Committee, ISAAC Phase Three Study Group, ISAAC International Data Centre. ISAAC: International Study of Asthma and Allergies in Childhood: phase three manual. Auckland, New Zealand: ISAAC International Data Centre; 2000.

Embassy of the United States - New Delhi, United States Environmental Protection Agency. United States Mission India NowCast Air Quality Data 2014. New Delhi, India: Embassy of the United States.

Euromonitor International. Euromonitor Passport - Cigarette Statistics 1999-2016. London, United Kingdom: Euromonitor International.

European Community Respiratory Health Survey Study Group. European Community Respiratory Health Survey 1992-2003 as provided by the Global Burden of Disease 2010 Respiratory Diseases Expert Group. [Data shared for this analysis]

Flohr C, Weiland SK, Weinmayr G, Björkstén B, Bråbäck L, Brunekreef B, Büchele G, Clausen M, Cookson WOC, von Mutius E, Strachan DP, Williams HC, ISAAC Phase Two Study Group. The role of atopic sensitization in flexural eczema: findings from the International Study of Asthma and Allergies in Childhood Phase Two. J Allergy Clin Immunol. 2008; 121(1): 141-147.

Flohr C, Weinmayr G, Weiland SK, Addo-Yobo E, Annesi-Maesano I, Björkstén B, Bråbäck L, Büchele G, Chico M, Cooper P, Clausen M, El Sharif N, Martinez Gimeno A, Mathur RS, von Mutius E, Morales Suarez-Varela M, Pearce N, Svabe V, Wong GWK, Yu M, Zhong NS, Williams HC, ISAAC Phase Two Study Group. How well do questionnaires perform compared with physical examination in detecting flexural eczema? Findings from the International Study of Asthma and Allergies in Childhood (ISAAC) Phase Two. Br J Dermatol. 2009; 161(4): 846-53.

Food and Agriculture Organization of the United Nations (FAO). FAOSTAT Commodity Balances - Crops Primary Equivalent. Rome, Italy: FAO.

Garcia-Marcos L, Robertson CF, Ross Anderson H, Ellwood P, Williams HC, Wong GW. Does migration affect asthma, rhinoconjunctivitis and eczema prevalence? Global findings from the international study of asthma and allergies in childhood. Int J Epidemiol. 2014; 43(6): 1846-54.

Global Burden of Disease Health Financing Collaborator Network, Institute for Health Metrics and Evaluation (IHME). Global Development Assistance for Health, Government, Prepaid Private, and Out-of-Pocket Health Spending 1995-2014. Seattle, United States: IHME; 2017.

Goudar SS, Goco N, Somannavar MS, Vernekar SS, Mallapur AA, Moore JL, Wallace DD, Sloan NL, Patel A, Hibberd PL, Koso-Thomas M, McClure EM, Goldenberg RL. Institutional deliveries and perinatal and neonatal mortality in Southern and Central India. Reprod Health. 2015; 12(Suppl 2): S13.

Guddattu V, Swathi A, Nair NS. Household and environment factors associated with asthma among Indian women: a multilevel approach. J Asthma. 2010; 47(4): 407-11.

Gupta R, Deedwania PC, Achari V, Asirvatham AJ, Bhansali A, Gupta A, Gupta B, Gupta S, Jali MV, Mahanta TG, Maheshwari A, Saboo B, Singh J. India Heart Watch Study 2005-2009. [Data shared for this analysis]

Gupta R. India - Jaipur Heart Watch Study Data on Blood Pressure, Cholesterol, BMI, and Fasting Blood Glucose 1993-2001. [Data shared for this analysis]

Healis-Sekhsaria Institute for Public Health, Madhya Pradesh Voluntary Health Association, Cancer Foundation of India, School of Preventive Oncology, University of Waterloo. International Tobacco Control Policy Evaluation Project: India Tobacco Control Survey 2010-2011. Navi Mumbai, India and Waterloo, Canada: Healis-Sekhsaria Institute for Public Health and University of Waterloo.

Health Management Information System (HMIS), National Health Mission, Ministry of Health and Family Welfare, Government of India. India Performance Related to Immunisation 2007-2009. New Delhi, India: Ministry of Health and Family Welfare.

Health Management Information System (HMIS), National Health Mission, Ministry of Health and Family Welfare, Government of India. India Performance Related to Immunisation 2008-2010. New Delhi, India: Ministry of Health and Family Welfare.

Health Management Information System (HMIS), National Health Mission, Ministry of Health and Family Welfare, Government of India. India Performance Related to Immunisation 2009-2011. New Delhi, India: Ministry of Health and Family Welfare.

Health Management Information System (HMIS), National Health Mission, Ministry of Health and Family Welfare, Government of India. India Performance Related to Immunisation 2010-2012. New Delhi, India: Ministry of Health and Family Welfare.

Health Management Information System (HMIS), National Health Mission, Ministry of Health and Family Welfare, Government of India. India Performance Related to Immunisation 2011-2013. New Delhi, India: Ministry of Health and Family Welfare.

Health Management Information System (HMIS), National Health Mission, Ministry of Health and Family Welfare, Government of India. India Performance Related to Immunisation 2012-2014. New Delhi, India: Ministry of Health and Family Welfare.

Health Management Information System (HMIS), National Health Mission, Ministry of Health and Family Welfare, Government of India. India Performance Related to Immunisation 2013-2015. New Delhi, India: Ministry of Health and Family Welfare.

Health Management Information System (HMIS), National Health Mission, Ministry of Health and Family Welfare, Government of India. India Performance Related to Immunisation 2014-2016. New Delhi, India: Ministry of Health and Family Welfare.

Health Management Information System (HMIS), National Health Mission, Ministry of Health and Family Welfare, Government of India. India Performance Related to Immunisation 2015-2017. New Delhi, India: Ministry of Health and Family Welfare.

Hemalatha R, Swetha GK, Seshacharyulu M, Radhakrishna KV. Respiratory syncitial virus in children with acute respiratory infections. Indian J Pediatr. 2010; 77(7): 755-8.

India Ground Monitor Station PM2.5 Data 2012 as it appears in World Health Organization (WHO). WHO Urban Ambient Air Pollution Database Draft 2016.

Indian Council of Medical Research (ICMR). India Study on Causes of Death by Verbal Autopsy 2003. New Delhi, India: ICMR. [Data shared for this analysis]

Indian Council of Medical Research, World Health Organization. India STEPS Noncommunicable Disease Risk Factors Survey 2003-2005. [Data shared for this analysis]

Institute of Health Systems, World Health Organization (WHO). WHO Multi-country Survey Study Report on Health and Health System Responsiveness, Andhra Pradesh 2000-2001.

Institute of Social Medicine and Health Policy, Shandong University, Shandong University School of Medicine, World Health Organization (WHO). China WHO Multi-country Survey Study on Health and Health System Responsiveness 2000-2001.

International Institute for Population Sciences (IIPS), Ministry of Health and Family Welfare, Government of India, Harvard T. H. Chan School of Public Health, RAND Corporation, Monash University, University of California Los Angeles, Indian Academy of Geriatrics, National AIDS Research Institute, Columbia University. Longitudinal Aging Study in India, Pilot Data 2010. Mumbai, India: IIPS.

International Institute for Population Sciences (IIPS), Ministry of Health and Family Welfare, Government of India, Macro International. India National Family Health Survey Data (NFHS-1) 1992-1993. Mumbai, India: IIPS.

International Institute for Population Sciences (IIPS), Ministry of Health and Family Welfare, Government of India, ORC Macro. India National Family Health Survey Data (NFHS-2) 1998-1999. Mumbai, India: IIPS.

International Institute for Population Sciences (IIPS), Ministry of Health and Family Welfare, Government of India, ORC Macro. India National Family Health Survey (NFHS-2) 1998-1999: National and State Reports. Mumbai, India: IIPS.

International Institute for Population Sciences (IIPS), Ministry of Health and Family Welfare, Government of India, Macro International. India National Family Health Survey Data (NFHS-3) 2005-2006. Mumbai, India: IIPS.

International Institute for Population Sciences (IIPS), Ministry of Health and Family Welfare, Government of India, Macro International. India National Family Health Survey (NFHS-3) 2005-2006: National and State Reports. Mumbai, India: IIPS.

International Institute for Population Sciences (IIPS), Ministry of Health and Family Welfare, Government of India, ICF. India National Family Health Survey (NFHS-4) 2015-2016: National and State-level Factsheets. Mumbai, India: IIPS.

International Institute for Population Sciences (IIPS), Ministry of Health and Family Welfare, Government of India. India District Level Household Survey Data (DLHS-1) 1998-1999. Mumbai, India: IIPS.

International Institute for Population Sciences (IIPS), Ministry of Health and Family Welfare, Government of India. India District Level Household Survey (DLHS-1) 1998-1999: National Report. Mumbai, India: IIPS.

International Institute for Population Sciences (IIPS), Ministry of Health and Family Welfare, Government of India. India District Level Household Survey Data (DLHS-2) 2002-2004. Mumbai, India: IIPS.

International Institute for Population Sciences (IIPS), Ministry of Health and Family Welfare, Government of India. India District Level Household Survey (DLHS-2) 2002-2004: National and State Reports. Mumbai, India: IIPS.

International Institute for Population Sciences (IIPS), Ministry of Health and Family Welfare, Government of India. India District Level Household and Facility Survey Data (DLHS-3) 2007-2008. Mumbai, India: IIPS.

International Institute for Population Sciences (IIPS), Ministry of Health and Family Welfare, Government of India. India District Level Household and Facility Survey (DLHS-3) 2007-2008: National and State Reports. Mumbai, India: IIPS.

International Institute for Population Sciences, Ministry of Health and Family Welfare, Government of India. India District Level Household and Facility Survey Data (DLHS-4) 2012-2013. Mumbai, India: IIPS.

International Institute for Population Sciences, World Health Organization (WHO). India WHO Study on Global Ageing and Adult Health 2007-2008.

International Institute for Population Sciences, World Health Organization. India World Health Survey 2003.

International Labour Organization (ILO). International Labour Statistics Database (ILOSTAT) - Employment by Sex and Economic Activity. Geneva, Switzerland: ILO.

International Social Survey Programme (ISSP), GESIS - Leibniz Institute of Social Sciences. ISSP: Family and Changing Gender Roles IV, ZA5900 (Version 4.0.0). Mannheim, Germany: GESIS - Leibniz Institute of Social Sciences; 2012.

Jain A, Vinod Bhat H, Acharya D. Prevalence of bronchial asthma in rural Indian children: a cross sectional study from South India. Indian J Pediatr. 2010; 77(1): 31-5.

Jindal SK, Aggarwal AN, Gupta D, Agarwal R, Kumar R, Kaur T, Chaudhry K, Shah B. Indian study on epidemiology of asthma, respiratory symptoms and chronic bronchitis in adults (INSEARCH). Int J Tuberc Lung Dis. 2012; 16(9): 1270-7.

John TJ, Cherian T, Steinhoff MC, Simoes EA, John M. Etiology of acute respiratory infections in children in tropical southern India. Rev Infect Dis. 1991; 13 Suppl 6: S463-9.

Johnson P, Balakrishnan K, Ramaswamy P, Ghosh S, Sadhasivam M, Abirami O, et al. Prevalence of chronic obstructive pulmonary disease in rural women of Tamilnadu: implications for refining disease burden assessments attributable to household biomass combustion. Glob Health Action. 2011;4(1):7226.

Joshi R, Cardona M, Iyengar S, Sukumar A, Raju CR, Raju KR, Raju K, Reddy KS, Lopez A, Neal B. Chronic diseases now a leading cause of death in rural India – mortality data from the Andhra Pradesh Rural Health Initiative. Int J Epidemiol. 2006; 35(6): 1522-9.

JSS Medical College and Hospital-Mysore, Population Health and Occupational Disease, National Heart & Lung Institute, Imperial College London. India - Mysore Burden of Obstructive Lung Disease Initiative Survey (BOLD) 2011-2014. [Data shared for this analysis]

Khandait DW, Ambadekar NN, Zodpey SP, Vasudeo ND. Maternal age as a risk factor for stillbirth. Indian J Public Health. 2000; 44(1): 28-30.

Kodkany BS, Derman RJ, Honnungar NV, Tyagi NK, Goudar SS, Mastiholi SC, Moore JL, McClure EM, Sloan N, Goldenberg RL. Establishment of a Maternal Newborn Health Registry in the Belgaum District of Karnataka, India. Reprod Health. 2015; 12 Suppl 2: S3.

Lai CK, Beasley R, Crane J, Foliaki S, Shah J, Weiland S. Global variation in the prevalence and severity of asthma symptoms: phase three of the International Study of Asthma and Allergies in Childhood (ISAAC). Thorax. 2009; 64(6): 476-483.

Mahesh PA, Jayaraj BS, Chaya SK, Lokesh KS, McKay AJ, Prabhakar AK, Pape UJ. Variation in the prevalence of chronic bronchitis among smokers: a cross-sectional study. Int J Tuberc Lung Dis. 2014; 18(7): 862-9.

Max Planck Institute for Demographic Research, Vienna Institute of Demography. Human Fertility Collection - Total Fertility Rate and Mean Age at Birth. Rostock, Germany: Max Planck Institute for Demographic Research.

McClure EM, Pasha O, Goudar SS, Chomba E, Garces A, Tshefu A, Althabe F, Esamai F, Patel A, Wright LL, Moore J, Kodkany BS, Belizan JM, Saleem S, Derman RJ, Carlo WA, Hambidge KM, Buekens P, Liechty EA, Bose C, Koso-Thomas M, Jobe AH, Goldenberg RL; Global Network Investigators. Epidemiology of stillbirth in low-middle income countries: A Global Network Study. Acta Obstet Gynecol Scand. 2011; 90(12): 1379-85.

Ministry of Health and Family Welfare, Government of India, International Institute for Population Sciences, Centers for Disease Control and Prevention (CDC), Johns Hopkins Bloomberg School of Public Health, Research Triangle Institute, Inc., World Health Organization. India Global Adult Tobacco Survey 2009-2010. Atlanta, United States: CDC.

Ministry of Health and Family Welfare, Government of India, United Nations Children's Fund. India Coverage Evaluation Survey 2005.

Ministry of Health and Family Welfare, Government of India, United Nations Children's Fund. India Coverage Evaluation Survey 2007.

Ministry of Health and Family Welfare, Government of India, United Nations Children's Fund. India Coverage Evaluation Survey Data 2009-2010. [Data shared for this analysis]

Ministry of Health and Family Welfare, Government of India, World Health Organization - Regional Office for South-East Asia and Country Office for India, Centers for Disease Control and Prevention. India-Kerala Global Youth Tobacco Survey 2004. New Delhi, India: Ministry of Health and Family Welfare.

Ministry of Health and Family Welfare, Government of India, World Health Organization - Regional Office for South-East Asia and Country Office for India, Centers for Disease Control and Prevention. India-Tripura Global Youth Tobacco Survey 2000. New Delhi, India: Ministry of Health and Family Welfare.

Ministry of Health and Family Welfare, Government of India, World Health Organization - Regional Office for South-East Asia and Country Office for India, Centers for Disease Control and Prevention. India-Uttar Pradesh Global Youth Tobacco Survey 2002. New Delhi, India: Ministry of Health and Family Welfare.

Ministry of Health and Family Welfare, Government of India, World Health Organization - Regional Office for South-East Asia and Country Office for India, Centers for Disease Control and Prevention. India-Uttaranchal Global Youth Tobacco Survey 2002. New Delhi, India: Ministry of Health and Family Welfare.

Ministry of Health and Family Welfare, Government of India, World Health Organization - Regional Office for South-East Asia and Country Office for India, Centers for Disease Control and Prevention. India-West Bengal Global Youth Tobacco Survey 2000. New Delhi, India: Ministry of Health and Family Welfare.

Ministry of Health and Family Welfare, Government of India, World Health Organization - Regional Office for South-East Asia and Country Office for India, Centers for Disease Control and Prevention. India Global Youth Tobacco Survey 2006. New Delhi, India: Ministry of Health and Family Welfare.

Ministry of Health and Family Welfare, Government of India, World Health Organization - Regional Office for South-East Asia and Country Office for India, Centers for Disease Control and Prevention. India Global Youth Tobacco Survey 2009. New Delhi, India: Ministry of Health and Family Welfare.

Ministry of Health and Family Welfare, Government of India, World Health Organization - Regional Office for South-East Asia and Country Office for India, Centers for Disease Control and Prevention. India-Arunachal Pradesh Global Youth Tobacco Survey 2000. New Delhi, India: Ministry of Health and Family Welfare.
Ministry of Health and Family Welfare, Government of India, World Health Organization - Regional Office for South-East Asia and Country Office for India, Centers for Disease Control and Prevention. India-Assam Global Youth Tobacco Survey 2000. New Delhi, India: Ministry of Health and Family Welfare.
Ministry of Health and Family Welfare, Government of India, World Health Organization - Regional Office for South-East Asia and Country Office for India, Centers for Disease Control and Prevention. India-Bihar Global Youth Tobacco Survey 2000. New Delhi, India: Ministry of Health and Family Welfare.
Ministry of Health and Family Welfare, Government of India, World Health Organization - Regional Office for South-East Asia and Country Office for India, Centers for Disease Control and Prevention. India-Goa Global Youth Tobacco Survey 2000. NNew Delhi, India: Ministry of Health and Family Welfare.
Ministry of Health and Family Welfare, Government of India, World Health Organization - Regional Office for South-East Asia and Country Office for India, Centers for Disease Control and Prevention. India-Gujarat Global Youth Tobacco Survey 2004. New Delhi, India: Ministry of Health and Family Welfare.
Ministry of Health and Family Welfare, Government of India, World Health Organization - Regional Office for South-East Asia and Country Office for India, Centers for Disease Control and Prevention. India-Gujarat with Ahmedabad Global Youth Tobacco Survey 2004. New Delhi, India: Ministry of Health and Family Welfare.
Ministry of Health and Family Welfare, Government of India, World Health Organization - Regional Office for South-East Asia and Country Office for India, Centers for Disease Control and Prevention. India-Haryana Global Youth Tobacco Survey 2004. New Delhi, India: Ministry of Health and Family Welfare.
Ministry of Health and Family Welfare, Government of India, World Health Organization - Regional Office for South-East Asia and Country Office for India, Centers for Disease Control and Prevention. India-Jammu and Kashmir Global Youth Tobacco Survey 2004. New Delhi, India: Ministry of Health and Family Welfare.
Ministry of Health and Family Welfare, Government of India, World Health Organization - Regional Office for South-East Asia and Country Office for India, Centers for Disease Control and Prevention. India-Karnataka Global Youth Tobacco Survey 2003. New Delhi, India: Ministry of Health and Family Welfare.
Ministry of Health and Family Welfare, Government of India, World Health Organization - Regional Office for South-East Asia and Country Office for India, Centers for Disease Control and Prevention. India-Madhya Pradesh Global Youth Tobacco Survey 2004. New Delhi, India: Ministry of Health and Family Welfare.
Ministry of Health and Family Welfare, Government of India, World Health Organization - Regional Office for South-East Asia and Country Office for India, Centers for Disease Control and Prevention. India-Maharashtra Global Youth Tobacco Survey 2000. New Delhi, India: Ministry of Health and Family Welfare.
Ministry of Health and Family Welfare, Government of India, World Health Organization - Regional Office for South-East Asia and Country Office for India, Centers for Disease Control and Prevention. India-Manipur Global Youth Tobacco Survey 2000. New Delhi, India: Ministry of Health and Family Welfare.
Ministry of Health and Family Welfare, Government of India, World Health Organization - Regional Office for South-East Asia and Country Office for India, Centers for Disease Control and Prevention. India-Meghalaya Global Youth Tobacco Survey 2000. New Delhi, India: Ministry of Health and Family Welfare.
Ministry of Health and Family Welfare, Government of India, World Health Organization - Regional Office for South-East Asia and Country Office for India, Centers for Disease Control and Prevention. India-Mizoram Global Youth Tobacco Survey 2000. New Delhi, India: Ministry of Health and Family Welfare.
Ministry of Health and Family Welfare, Government of India, World Health Organization - Regional Office for South-East Asia and Country Office for India, Centers for Disease Control and Prevention. India-Nagaland Global Youth Tobacco Survey 2000. New Delhi, India: Ministry of Health and Family Welfare.
Ministry of Health and Family Welfare, Government of India, World Health Organization - Regional Office for South-East Asia and Country Office for India, Centers for Disease Control and Prevention. India-Punjab Global Youth Tobacco Survey 2004. New Delhi, India: Ministry of Health and Family Welfare.
Ministry of Health and Family Welfare, Government of India, World Health Organization - Regional Office for South-East Asia and Country Office for India, Centers for Disease Control and Prevention. India-Rajasthan Global Youth Tobacco Survey 2003. New Delhi, India: Ministry of Health and Family Welfare.
Ministry of Health and Family Welfare, Government of India, World Health Organization - Regional Office for South-East Asia and Country Office for India, Centers for Disease Control and Prevention. India-Sikkim Global Youth Tobacco Survey 2000. New Delhi, India: Ministry of Health and Family Welfare.
Ministry of Health and Family Welfare, Government of India, World Health Organization - Regional Office for South-East Asia and Country Office for India, Centers for Disease Control and Prevention. India-Tamil Nadu Global Youth Tobacco Survey 2000. New Delhi, India: Ministry of Health and Family Welfare.

Ministry of Statistics and Programme Implementation, Government of India. Consumer Expenditure Survey, National Sample Survey Round 38, January-December 1983. New Delhi, India: Ministry of Statistics and Programme Implementation.

Ministry of Statistics and Programme Implementation, Government of India. Employment and Unemployment Survey, National Sample Survey Round 38, January-December 1983. New Delhi, India: Ministry of Statistics and Programme Implementation.

Ministry of Statistics and Programme Implementation, Government of India. Survey on Participation in Education, National Sample Survey Round 42, July 1986- June 1987. New Delhi, India: Ministry of Statistics and Programme Implementation.

Ministry of Statistics and Programme Implementation, Government of India. Consumer Expenditure Survey, National Sample Survey Round 43, July 1987-June 1988. New Delhi, India: Ministry of Statistics and Programme Implementation.

Ministry of Statistics and Programme Implementation, Government of India. Employment and Unemployment Survey, National Sample Survey Round 43, July 1987- June 1988. New Delhi, India: Ministry of Statistics and Programme Implementation.

Ministry of Statistics and Programme Implementation, Government of India. Consumer Expenditure Survey, National Sample Survey Round 47, July - December 1991. New Delhi, India: Ministry of Statistics and Programme Implementation.

Ministry of Statistics and Programme Implementation, Government of India. Survey on Literacy and Culture, National Sample Survey Round 47, July - December 1991. New Delhi, India: Ministry of Statistics and Programme Implementation.

Ministry of Statistics and Programme Implementation, Government of India. Consumer Expenditure Survey, National Sample Survey Round 48, January-December 1992. New Delhi, India: Ministry of Statistics and Programme Implementation.

Ministry of Statistics and Programme Implementation, Government of India. Consumer Expenditure Survey, National Sample Survey Round 49, January-June 1993. New Delhi, India: Ministry of Statistics and Programme Implementation.

Ministry of Statistics and Programme Implementation, Government of India. Consumer Expenditure Survey, National Sample Survey Round 50, July 1993-June 1994. New Delhi, India: Ministry of Statistics and Programme Implementation.

Ministry of Statistics and Programme Implementation, Government of India. Employment and Unemployment Survey, National Sample Survey Round 50, July 1993- June 1994. New Delhi, India: Ministry of Statistics and Programme Implementation.

Ministry of Statistics and Programme Implementation, Government of India. Consumer Expenditure Survey, National Sample Survey Round 52, July 1995-June 1996. New Delhi, India: Ministry of Statistics and Programme Implementation.

Ministry of Statistics and Programme Implementation, Government of India. Participation in Education Survey, National Sample Survey Round 52, July 1995-June 1996. New Delhi, India: Ministry of Statistics and Programme Implementation.

Ministry of Statistics and Programme Implementation, Government of India. Survey on Healthcare, National Sample Survey Round 52, July 1995-June 1996. New Delhi, India: Ministry of Statistics and Programme Implementation.

Ministry of Statistics and Programme Implementation, Government of India. Consumer Expenditure Survey, India National Sample Survey Round 55, July 1999-June 2000. New Delhi, India: Ministry of Statistics and Programme Implementation.

Ministry of Statistics and Programme Implementation, Government of India. Employment and Unemployment Survey, India National Sample Survey Round 55, July 1999-June 2000. New Delhi, India: Ministry of Statistics and Programme Implementation.

Ministry of Statistics and Programme Implementation, Government of India. Consumer Expenditure Survey, National Sample Survey Round 56, July 2000 - June 2001. New Delhi, India: Ministry of Statistics and Programme Implementation.

Ministry of Statistics and Programme Implementation, Government of India. Consumer Expenditure Survey, National Sample Survey Round 60, January-June 2004. New Delhi, India: Ministry of Statistics and Programme Implementation.

Ministry of Statistics and Programme Implementation, Government of India. Employment and Unemployment surveys, National Sample Survey Round 60, January-June 2004. New Delhi, India: Ministry of Statistics and Programme Implementation.

Ministry of Statistics and Programme Implementation, Government of India. Survey on Morbidity and Healthcare, National Sample Survey Round 60, January-June 2004. New Delhi, India: Ministry of Statistics and Programme Implementation.

Ministry of Statistics and Programme Implementation, Government of India. Consumer Expenditure Survey, National Sample Survey Round 61, July 2004 - June 2005. New Delhi, India: Ministry of Statistics and Programme Implementation.

Ministry of Statistics and Programme Implementation, Government of India. Employment and Unemployment Survey, National Sample Survey Round 61, July 2004- June 2005. New Delhi, India: Ministry of Statistics and Programme Implementation.

Ministry of Statistics and Programme Implementation, Government of India. Consumer Expenditure Survey, National Sample Survey Round 63, July 2006- June 2007. New Delhi, India: Ministry of Statistics and Programme Implementation.

Ministry of Statistics and Programme Implementation, Government of India. Consumer Expenditure Survey, National Sample Survey Round 64, July 2007- June 2008. New Delhi, India: Ministry of Statistics and Programme Implementation.

Ministry of Statistics and Programme Implementation, Government of India. Employment and Unemployment Survey, National Sample Survey Round 64, July 2007- June 2008. New Delhi, India: Ministry of Statistics and Programme Implementation.

Ministry of Statistics and Programme Implementation, Government of India. Participation & Expenditure in Education Survey, National Sample Survey Round 64, July 2007- June 2008. New Delhi, India: Ministry of Statistics and Programme Implementation.

Ministry of Statistics and Programme Implementation, Government of India. Consumer Expenditure Survey, National Sample Survey Round 66, July 2009-June 2010. New Delhi, India: Ministry of Statistics and Programme Implementation.

Ministry of Statistics and Programme Implementation, Government of India. Employment and Unemployment Survey, National Sample Survey Round 66, July 2009-June 2010. New Delhi, India: Ministry of Statistics and Programme Implementation.

Ministry of Statistics and Programme Implementation, Government of India. Consumer Expenditure Survey, National Sample Survey Round 68, July 2011- June 2012. New Delhi, India: Ministry of Statistics and Programme Implementation.
Ministry of Statistics and Programme Implementation, Government of India. Employment and Unemployment Survey, National Sample Survey Round 68, July 2011- June 2012. New Delhi, India: Ministry of Statistics and Programme Implementation.
Ministry of Statistics and Programme Implementation, Government of India. Social Consumption Survey on Health and Education, National Sample Survey Round 71, January 2014 - June 2014. New Delhi, India: Ministry of Statistics and Programme Implementation.
Ministry of Statistics and Programme Implementation, Government of India. Household expenditure on services and durable goods survey, National Sample Survey Round 72, July 2014 - June 2015. New Delhi, India: Ministry of Statistics and Programme Implementation.
Ministry of Statistics and Programme Implementation, Government of India. India State Series: Gross State Domestic Product at Factor Cost by Industry of Origin Tables 1980-81 to 1993-94. New Delhi, India: Ministry of Statistics and Programme Implementation.
Ministry of Statistics and Programme Implementation, Government of India. India State Series: Gross State Domestic Product at Factor Cost by Industry of Origin Tables 1993-94 to 2004-05. New Delhi, India: Ministry of Statistics and Programme Implementation.
Ministry of Statistics and Programme Implementation, Government of India. India State Series: Gross State Domestic Product at Factor Cost by Industry of Origin Tables 1999-2000 to 2009-10. New Delhi, India: Ministry of Statistics and Programme Implementation.
Ministry of Statistics and Programme Implementation, Government of India. India State Series: Gross State Domestic Product at Factor Cost by Industry of Origin Tables 2004-05 to 2013-14. New Delhi, India: Ministry of Statistics and Programme Implementation.
Mony PK, Varghese B, Thomas T. Estimation of perinatal mortality rate for institutional births in Rajasthan state, India, using capture-recapture technique. BMJ Open. 2015; 5(3): e005966.
Nagel G, Weinmayr G, Flohr C, Kleiner A, Strachan DP. Association of pertussis and measles infections and immunizations with asthma and allergic sensitization in ISAAC Phase Two. Pediatr Allergy Immunol. 2012; 23(8): 737-46.
Narayana P, Prasanna M, Narahari S, Guruprasad A. Prevalence of asthma in school children in rural India. Ann Thorac Med. 2010;5(2):118.
National Nutrition Monitoring Bureau, National Institute of Nutrition (NIN), Indian Council of Medical Research. India Rural Survey of Diet and Nutritional Status Data 2004-2006. [Data shared for this analysis]
National Nutrition Monitoring Bureau, National Institute of Nutrition (NIN), Indian Council of Medical Research. India Rural Survey of Diet and Nutritional Status Report 2004-2006. Hyderabad, India: NIN.
National Institute of Medical Statistics, Indian Council of Medical Research (ICMR), Integrated Disease Surveillance Programme. Non-Communicable Disease Risk Factors Survey Data 2007-2008. New Delhi, India: ICMR. [Data shared for this analysis]
National Nutrition Monitoring Bureau, National Institute of Nutrition (NIN), Indian Council of Medical Research. India Rural Third Repeat Survey of Diet and Nutritional Status Data 2011-2012. [Data shared for this analysis]
National Nutrition Monitoring Bureau, National Institute of Nutrition (NIN), Indian Council of Medical Research. India Rural Third Repeat Survey of Diet and Nutritional Status Report 2011-2012. Hyderabad, India: NIN.
National Nutrition Monitoring Bureau, National Institute of Nutrition (NIN), Indian Council of Medical Research. India Tribal Second Repeat Survey of Diet and Nutritional Status Data 2007-2008. [Data shared for this analysis]
National Nutrition Monitoring Bureau, National Institute of Nutrition (NIN), Indian Council of Medical Research. India Tribal Second Repeat Survey of Diet and Nutritional Status Report 2007-2008. Hyderabad, India: NIN.
Nazareth Hospital. Inpatient and Discharge Data 2014. Shillong, Meghalaya: Nazareth Hospital. [Data shared for this analysis]
Office of the Registrar General & Census Commissioner, Centre for Global Health Research - University of Toronto. India SRS Special Fertility and Mortality Survey 1998. New Delhi, India: Office of the Registrar General & Census Commissioner; 2005.
Office of the Registrar General & Census Commissioner, Ministry of Home Affairs, Government of India. India Annual Health Survey Data 2010-2011. New Delhi, India: Office of the Registrar General & Census Commissioner.
Office of the Registrar General & Census Commissioner, Ministry of Home Affairs, Government of India. India Annual Health Survey Report 2010-2011. New Delhi, India: Office of the Registrar General & Census Commissioner.
Office of the Registrar General & Census Commissioner, Ministry of Home Affairs, Government of India. India Annual Health Survey Data 2011-2012. New Delhi, India: Office of the Registrar General & Census Commissioner.
Office of the Registrar General & Census Commissioner, Ministry of Home Affairs, Government of India. India Annual Health Survey Report 2011-2012. New Delhi, India: Office of the Registrar General & Census Commissioner.
Office of the Registrar General & Census Commissioner, Ministry of Home Affairs, Government of India. India Annual Health Survey Data 2012-2013. New Delhi, India: Office of the Registrar General & Census Commissioner.
Office of the Registrar General & Census Commissioner, Ministry of Home Affairs, Government of India. India Annual Health Survey Report 2012-2013. New Delhi, India: Office of the Registrar General & Census Commissioner.
Office of the Registrar General & Census Commissioner, Ministry of Home Affairs, Government of India. India Annual Health Survey Data 2010-2013. New Delhi, India: Office of the Registrar General & Census Commissioner.
Office of the Registrar General & Census Commissioner, Ministry of Home Affairs, Government of India. India Annual Health Survey Report 2010-2013. New Delhi, India: Office of the Registrar General & Census Commissioner.

Office of the Registrar General & Census Commissioner, Ministry of Home Affairs, Government of India. India Medical Certification of Cause of Death Report 1997. New Delhi, India: Office of the Registrar General & Census Commissioner.
Office of the Registrar General & Census Commissioner, Ministry of Home Affairs, Government of India. India Medical Certification of Cause of Death Report 2005. New Delhi, India: Office of the Registrar General & Census Commissioner.
Office of the Registrar General & Census Commissioner, Ministry of Home Affairs, Government of India. India Medical Certification of Cause of Death Report 2006. New Delhi, India: Office of the Registrar General & Census Commissioner.
Office of the Registrar General & Census Commissioner, Ministry of Home Affairs, Government of India. India Medical Certification of Cause of Death Report 2008. New Delhi, India: Office of the Registrar General & Census Commissioner.
Office of the Registrar General & Census Commissioner, Ministry of Home Affairs, Government of India. India Medical Certification of Cause of Death Report 2009. New Delhi, India: Office of the Registrar General & Census Commissioner; 2014.
Office of the Registrar General & Census Commissioner, Ministry of Home Affairs, Government of India. India Medical Certification of Cause of Death Report 2010. New Delhi, India: Office of the Registrar General & Census Commissioner; 2014.
Office of the Registrar General & Census Commissioner, Ministry of Home Affairs, Government of India. India Medical Certification of Cause of Death State-Level Tabulations 1990. New Delhi, India: Office of the Registrar General & Census Commissioner.
Office of the Registrar General & Census Commissioner, Ministry of Home Affairs, Government of India. India Medical Certification of Cause of Death State-Level Tabulations 1991. New Delhi, India: Office of the Registrar General & Census Commissioner.
Office of the Registrar General & Census Commissioner, Ministry of Home Affairs, Government of India. India Medical Certification of Cause of Death State-Level Tabulations 1992. New Delhi, India: Office of the Registrar General & Census Commissioner.
Office of the Registrar General & Census Commissioner, Ministry of Home Affairs, Government of India. India Medical Certification of Cause of Death State-Level Tabulations 1993. New Delhi, India: Office of the Registrar General & Census Commissioner.
Office of the Registrar General & Census Commissioner, Ministry of Home Affairs, Government of India. India Medical Certification of Cause of Death State-Level Tabulations 1994. New Delhi, India: Office of the Registrar General & Census Commissioner.
Office of the Registrar General & Census Commissioner, Ministry of Home Affairs, Government of India. India Medical Certification of Cause of Death State-Level Tabulations 1995. New Delhi, India: Office of the Registrar General & Census Commissioner.
Office of the Registrar General & Census Commissioner, Ministry of Home Affairs, Government of India. India Medical Certification of Cause of Death State-Level Tabulations 1996. New Delhi, India: Office of the Registrar General & Census Commissioner.
Office of the Registrar General & Census Commissioner, Ministry of Home Affairs, Government of India. India Medical Certification of Cause of Death State-Level Tabulations 1998. New Delhi, India: Office of the Registrar General & Census Commissioner.
Office of the Registrar General & Census Commissioner, Ministry of Home Affairs, Government of India. India Medical Certification of Cause of Death State-Level Tabulations 1999. New Delhi, India: Office of the Registrar General & Census Commissioner.
Office of the Registrar General & Census Commissioner, Ministry of Home Affairs, Government of India. India Medical Certification of Cause of Death State-Level Tabulations 2000. New Delhi, India: Office of the Registrar General & Census Commissioner.
Office of the Registrar General & Census Commissioner, Ministry of Home Affairs, Government of India. India Medical Certification of Cause of Death State-Level Tabulations 2001. New Delhi, India: Office of the Registrar General & Census Commissioner.
Office of the Registrar General & Census Commissioner, Ministry of Home Affairs, Government of India. India Medical Certification of Cause of Death State-Level Tabulations 2002. New Delhi, India: Office of the Registrar General & Census Commissioner.
Office of the Registrar General & Census Commissioner, Ministry of Home Affairs, Government of India. India Medical Certification of Cause of Death State-Level Tabulations 2003. New Delhi, India: Office of the Registrar General & Census Commissioner.
Office of the Registrar General & Census Commissioner, Ministry of Home Affairs, Government of India. India Medical Certification of Cause of Death State-Level Tabulations 2004. New Delhi, India: Office of the Registrar General & Census Commissioner.
Office of the Registrar General & Census Commissioner, Ministry of Home Affairs, Government of India. India Population and Housing Census 1981. New Delhi, India: Office of the Registrar General & Census Commissioner.
Office of the Registrar General & Census Commissioner, Ministry of Home Affairs, Government of India. India Population and Housing Census 1991. New Delhi, India: Office of the Registrar General & Census Commissioner.
Office of the Registrar General & Census Commissioner, Ministry of Home Affairs, Government of India. India Population and Housing Census 2001. New Delhi, India: Office of the Registrar General & Census Commissioner.
Office of the Registrar General & Census Commissioner, Ministry of Home Affairs, Government of India. India Sample Registration System Baseline Survey 2014. New Delhi, India: Office of the Registrar General & Census Commissioner.
Office of the Registrar General & Census Commissioner, Ministry of Home Affairs, Government of India. India SRS Statistical Report 1999. New Delhi, India: Office of the Registrar General & Census Commissioner.
Office of the Registrar General & Census Commissioner, Ministry of Home Affairs, Government of India. India SRS Statistical Report 2000. New Delhi, India: Office of the Registrar General & Census Commissioner.
Office of the Registrar General & Census Commissioner, Ministry of Home Affairs, Government of India. India SRS Statistical Report 2001. New Delhi, India: Office of the Registrar General & Census Commissioner; 2005.

Office of the Registrar General & Census Commissioner, Ministry of Home Affairs, Government of India. India SRS Statistical Report 2002. New Delhi, India: Office of the Registrar General & Census Commissioner; 2005.
Office of the Registrar General & Census Commissioner, Ministry of Home Affairs, Government of India. India SRS Statistical Report 2003. New Delhi, India: Office of the Registrar General and Census Commissioner; 2005.
Office of the Registrar General & Census Commissioner, Ministry of Home Affairs, Government of India. India SRS Statistical Report 2004. New Delhi, India: Office of the Registrar General and Census Commissioner; 2006.
Office of the Registrar General & Census Commissioner, Ministry of Home Affairs, Government of India. India SRS Statistical Report 2005. New Delhi, India: Office of the Registrar General and Census Commissioner; 2006.
Office of the Registrar General & Census Commissioner, Ministry of Home Affairs, Government of India. India SRS Statistical Report 2006. New Delhi, India: Office of the Registrar General and Census Commissioner; 2008.
Office of the Registrar General & Census Commissioner, Ministry of Home Affairs, Government of India. India SRS Statistical Report 2007. New Delhi, India: Office of the Registrar General and Census Commissioner; 2008.
Office of the Registrar General & Census Commissioner, Ministry of Home Affairs, Government of India. India SRS Statistical Report 2008. New Delhi, India: Office of the Registrar General and Census Commissioner; 2010.
Office of the Registrar General & Census Commissioner, Ministry of Home Affairs, Government of India. India SRS Statistical Report 2009. New Delhi, India: Office of the Registrar General and Census Commissioner.
Office of the Registrar General & Census Commissioner, Ministry of Home Affairs, Government of India. India SRS Statistical Report 2010. New Delhi, India: Office of the Registrar General and Census Commissioner; 2012.
Office of the Registrar General & Census Commissioner, Ministry of Home Affairs, Government of India. India SRS Statistical Report 2011. New Delhi, India: Office of the Registrar General and Census Commissioner; 2013.
Office of the Registrar General & Census Commissioner, Ministry of Home Affairs, Government of India. India SRS Statistical Report 2012. New Delhi, India: Office of the Registrar General and Census Commissioner; 2013.
Office of the Registrar General & Census Commissioner, Ministry of Home Affairs, Government of India. India SRS Statistical Report 2013. New Delhi, India: Office of the Registrar General and Census Commissioner; 2014.
Office of the Registrar General & Census Commissioner, Ministry of Home Affairs, Government of India. India SRS Statistical Report 2014. New Delhi, India: Office of the Registrar General and Census Commissioner.
Office of the Registrar General & Census Commissioner, Ministry of Home Affairs, Government of India. India SRS Verbal Autopsy 2004-2006. New Delhi, India: Office of the Registrar General and Census Commissioner. [Data shared for this analysis]
Office of the Registrar General & Census Commissioner, Ministry of Home Affairs, Government of India. India SRS Verbal Autopsy Report 2004-2006. New Delhi, India: Office of the Registrar General and Census Commissioner.
Office of the Registrar General & Census Commissioner, Ministry of Home Affairs, Government of India. India SRS Verbal Autopsy 2007-2009. New Delhi, India: Office of the Registrar General and Census Commissioner. [Data shared for this analysis]
Office of the Registrar General & Census Commissioner, Ministry of Home Affairs, Government of India. India SRS Verbal Autopsy Report 2007-2009. New Delhi, India: Office of the Registrar General and Census Commissioner.
Office of the Registrar General & Census Commissioner, Ministry of Home Affairs, Government of India. India SRS Verbal Autopsy 2010-2013. New Delhi, India: Office of the Registrar General and Census Commissioner. [Data shared for this analysis]
Office of the Registrar General & Census Commissioner, Ministry of Home Affairs, Government of India. India SRS Verbal Autopsy Report 2010-2013. New Delhi, India: Office of the Registrar General and Census Commissioner.
Office of the Registrar General & Census Commissioner, Ministry of Home Affairs, Government of India. India Survey of Causes of Death 1980. New Delhi, India: Office of the Registrar General and Census Commissioner.
Office of the Registrar General & Census Commissioner, Ministry of Home Affairs, Government of India. India Survey of Causes of Death 1981. New Delhi, India: Office of the Registrar General and Census Commissioner.
Office of the Registrar General & Census Commissioner, Ministry of Home Affairs, Government of India. India Survey of Causes of Death 1982. New Delhi, India: Office of the Registrar General and Census Commissioner.
Office of the Registrar General & Census Commissioner, Ministry of Home Affairs, Government of India. India Survey of Causes of Death 1983. New Delhi, India: Office of the Registrar General and Census Commissioner.
Office of the Registrar General & Census Commissioner, Ministry of Home Affairs, Government of India. India Survey of Causes of Death 1984. New Delhi, India: Office of the Registrar General and Census Commissioner.
Office of the Registrar General & Census Commissioner, Ministry of Home Affairs, Government of India. India Survey of Causes of Death 1985. New Delhi, India: Office of the Registrar General and Census Commissioner.
Office of the Registrar General & Census Commissioner, Ministry of Home Affairs, Government of India. India Survey of Causes of Death 1986. New Delhi, India: Office of the Registrar General and Census Commissioner.
Office of the Registrar General & Census Commissioner, Ministry of Home Affairs, Government of India. India Vital Statistics 1980. New Delhi, India: Office of the Registrar General and Census Commissioner.
Office of the Registrar General & Census Commissioner, Ministry of Home Affairs, Government of India. India Vital Statistics 1981. New Delhi, India: Office of the Registrar General and Census Commissioner.

Office of the Registrar General & Census Commissioner, Ministry of Home Affairs, Government of India. India Vital Statistics 1982. New Delhi, India: Office of the Registrar General and Census Commissioner.
Office of the Registrar General & Census Commissioner, Ministry of Home Affairs, Government of India. India Vital Statistics 1983. New Delhi, India: Office of the Registrar General and Census Commissioner.
Office of the Registrar General & Census Commissioner, Ministry of Home Affairs, Government of India. India Vital Statistics 1984. New Delhi, India: Office of the Registrar General and Census Commissioner.
Office of the Registrar General & Census Commissioner, Ministry of Home Affairs, Government of India. India Vital Statistics 1986. New Delhi, India: Office of the Registrar General and Census Commissioner.
Office of the Registrar General & Census Commissioner, Ministry of Home Affairs, Government of India. India Vital Statistics 1987. New Delhi, India: Office of the Registrar General and Census Commissioner.
Office of the Registrar General & Census Commissioner, Ministry of Home Affairs, Government of India. India Vital Statistics 1989. New Delhi, India: Office of the Registrar General and Census Commissioner.
Parasuramalu BG, Huliraj N, Rudraprasad BM, Prashanth Kumar SP, Gangaboraiah, Ramesh Masthi NR. Prevalence of bronchial asthma and its association with smoking habits among adult population in rural area. Indian J Public Health. 2010; 54(3): 165-8.
Parihar YS, Patnaik JP, Nema BK, Sahoo GB, Misra IB, Adhikary S. Coal workers' pneumoconiosis: a study of prevalence in coal mines of eastern Madhya Pradesh and Orissa states of India. Ind Health. 1997; 35(4): 467-73.
Patwari AK, Bisht S, Srinivasan A, Deb M, Chattopadhya D. Aetiology of pneumonia in hospitalized children. J Trop Pediatr. 1996; 42(1): 15–20.
Pearce N, Ait-Khaled N, Beasley R, Mallol J, Keil U, Mitchell E, et al. Worldwide trends in the prevalence of asthma symptoms: phase III of the International Study of Asthma and Allergies in Childhood (ISAAC). Thorax. 2007;62(9):758–66.
Public Health Foundation of India. India Cause of Death Estimation Study in Bihar 2011-2014. New Delhi, India: Public Health Foundation of India. [Data shared for this analysis]
Saleem S, McClure EM, Goudar SS, Patel A, Esamai F, Garces A, Chomba E, Althabe F, Moore J, Kodkany B, Pasha O, Belizan J, Mayansyan A, Derman RJ, Hibberd PL, Liechty EA, Krebs NF, Hambidge KM, Buekens P, Carlo WA, Wright LL, Koso-Thomas M, Jobe AH, Goldenberg RL, on behalf of the Global Network Maternal Newborn Health Registry Study Investigators. A prospective study of maternal, fetal and neonatal deaths in low- and middle-income countries. Bull World Health Organ. 2014; 92(8): 605-12.
Sharma BS, Kumar MG, Chandel R. Prevalence of asthma in urban school children in Jaipur, Rajasthan. Indian Pediatr. 2012; 49(10): 835-6.
Sher-i-Kashmir Institute of Medical Sciences-Srinagar, Population Health and Occupational Disease, National Heart & Lung Institute, Imperial College London. India - Srinagar Burden of Obstructive Lung Disease Intiative Survey (BOLD) 2010-2011. [Data shared for this analysis]
Singh AK, Jain A, Jain B, Singh KP, Dangi T, Mohan M, Dwivedi M, Kumar R, Kushwaha R a. S, Singh JV, Mishra AC, Chhaddha MS. Viral aetiology of acute lower respiratory tract illness in hospitalised paediatric patients of a tertiary hospital: one year prospective study. Indian J Med Microbiol. 2014; 32(1): 13–8.
Singh S, Sharma BB, Sharma SK, Sabir M, Singh V, investigators I collaborating. Prevalence and severity of asthma among Indian school children aged between 6 and 14 years: associations with parental smoking and traffic pollution. J Asthma. 2016; 53(3): 238–44.
Sinha B, Singla R, Chowdhury R. An epidemiological profile of chronic obstructive pulmonary disease: A community-based study in Delhi. J Postgrad Med. 2016; 63(1):29.
Sinha B, Singla R, Chowdhury R. Community-based study on chronic obstructive pulmonary diseasey in Delhi dataset 2016. [Data shared for this analysis]
Spector JM, Agrawal P, Kodkany B, Lipsitz S, Lashoher A, Dziekan G, Bahl R, Merialdi M, Mathai M, Lemer C, Gawande A. Improving Quality of Care for Maternal and Newborn Health: Prospective Pilot Study of the WHO Safe Childbirth Checklist Program. PLoS One. 2012; 7(5): e35151.
Tiwari S, Hopke PK, Pipal AS, Srivastava AK, Bishta DS, Tiwari S, Singh AK, Sonie VK, Attrie SD. Intra-urban variability of particulate matter (PM2.5 and PM10) and its relationship with optical properties of aerosols over Delhi, India. Atmos Res. 2015; 166: 223-32.
Tobacco Sales Data. Personal Correspondence with Christopher Tan, 1997-2012. [Data shared for this analysis]
U.S. Department of Agriculture (USDA). USDA Global Tobacco Database 1960-2005. Washington DC , United States: USDA.
United States Geological Survey (USGS). USGS Mineral Industry Surveys: World Asbestos Consumption 2003-2007. Reston, United States: USGS; 2009.
United States Geological Survey (USGS). USGS Minerals Yearbook 2013. Reston, United States: USGS.
Virta RL, United States Geological Survey (USGS). Worldwide Asbestos Supply and Consumption Trends from 1990 through 2003: USGS Circular 1298. Reston, United States: USGS; 2006.
Vogel JP, Souza JP, Mori R, Morisaki N, Lumbiganon P, Laopaiboon M, Ortiz-Panozo E, Hernandez B, Pérez-Cuevas R, Roy M, Mittal S, Cecatti JG, Tunçalp Ö, Gülmezoglu AM; WHO Multicountry Survey on Maternal and Newborn Health Research Network. Maternal complications and perinatal mortality: findings of the World Health Organization Multicountry Survey on Maternal and Newborn Health. BJOG. 2014; 121(Supp 1): 76-88.
World Bank. Survey of Living Conditions 1997-1998, Uttar Pradesh and Bihar. Washington D.C., United States: World Bank.

World Energy Council. Survey of Energy Resources 2010. London, United Kingdom: World Energy Council; 2010.
World Health Organization (WHO), United Nations Children's Fund (UNICEF). WHO and UNICEF Reported Administrative Data for Immunization Coverage Time Series. Available from: http://www.who.int/immunization/monitoring_surveillance/data/en/
World Health Organization (WHO). WHO Global Survey on Maternal and Perinatal Health 2004-2008. Geneva, Switzerland: WHO.
World Health Organization (WHO). WHO Urban Ambient Air Pollution Database Draft 2016.
Worldwide variations in the prevalence of asthma symptoms: the International Study of Asthma and Allergies in Childhood (ISAAC). Eur Respir J. 1998; 12(2): 315-35.

3. Prevalent cases of and deaths from COPD and asthma in the states of India, 2016

States of India (population in 2016)	COPD		Asthma	
	Prevalent cases in thousands (95% uncertainty interval)	Deaths cases in thousands (95% uncertainty interval)	Prevalent cases in thousands (95% uncertainty interval)	Deaths cases in thousands (95% uncertainty interval)
**India (1,316 million)**	**55,320 (53,061 to 57,614)**	**848 (765 to 939)**	**37,867 (35,736 to 40,155)**	**183 (118 to 247)**
**Low ETL (626 million)**	**2,5121 (2,4073 to 2,6161)**	**261 (222 to 301)**	**17,184 (16,180 to 18,260)**	**55 (27 to 91)**
Bihar	3,831 (3,673 to 4,003)	60 (50 to 72)	2,882 (2,706 to 3,069)	14 (9 to 20)
Jharkhand	1,144 (1,095 to 1,195)	13 (10 to 17)	927 (870 to 986)	3 (2 to 5)
Uttar Pradesh	9,393 (8,984 to 9,792)	211 (178 to 245)	5,783 (5,398 to 6,187)	49 (30 to 70)
Rajasthan	3,404 (3,264 to 3,549)	84 (66 to 100)	2,391 (2,259 to 2,534)	18 (11 to 24)
Meghalaya	85 (81 to 90)	1 (1 to 1)	112 (107 to 118)	<1
Assam	1,328 (1,273 to 1,383)	22 (19 to 26)	975 (916 to 1,035)	5 (3 to 8)
Chhattisgarh	1,037 (991 to 1,082)	11 (9 to 16)	706 (658 to 757)	3 (1 to 5)
Madhya Pradesh	3,043 (2,915 to 3,173)	48 (40 to 57)	2,082 (1,957 to 2,216)	11 (7 to 16)
Odisha	1,855 (1,774 to 1,938)	17 (13 to 30)	1,326 (1,242 to 1,420)	4 (2 to 9)
**Lower-middle ETL (92 million)**	**3,889 (3,732 to 4,059)**	**34 (30 to 39)**	**2,727 (2,577 to 2,884)**	**6 (3 to 9)**
Arunachal Pradesh	42 (40 to 44)	<1	45 (43 to 48)	<1
Mizoram	57 (54 to 59)	1 (1 to 1)	35 (33 to 37)	<1
Nagaland	63 (60 to 65)	<1	98 (93 to 102)	<1
Uttarakhand	532 (510 to 554)	11 (9 to 12)	347 (324 to 370)	2 (1 to 3)
Gujarat	2,851 (2,735 to 2,976)	43 (37 to 48)	1,881 (1,770 to 2,001)	9 (6 to 12)
Tripura	202 (193 to 210)	3 (3 to 4)	210 (201 to 219)	1 (0 to 1)
Sikkim	23 (22 to 24)	<1	30 (29 to 31)	<1
Manipur	120 (115 to 126)	1 (1 to 2)	81 (75 to 87)	<1
**Higher-middle ETL (446 million)**	**19,622 (18,842 to 20,436)**	**138 (124 to 154)**	**13,458 (12,728 to 14,254)**	**23 (13 to 34)**
Haryana	1,401 (1,343 to 1,462)	23 (20 to 27)	804 (753 to 859)	4 (3 to 6)
Delhi	843 (814 to 872)	4 (4 to 5)	447 (414 to 481)	1 (1 to 1)
Telangana	1,604 (1,538 to 1,675)	19 (15 to 23)	1,131 (1,066 to 1,205)	4 (2 to 6)
Andhra Pradesh	2,301 (2,061 to 2,614)	29 (24 to 35)	1,919 (1,824 to 2,155)	6 (4 to 9)
Jammu and Kashmir	657 (634 to 680)	9 (8 to 11)	323 (304 to 344)	2 (1 to 2)
Karnataka	3,080 (2,960 to 3,207)	42 (36 to 48)	1,823 (1,709 to 1,941)	9 (6 to 13)
West Bengal	4,338 (4,161 to 4,528)	50 (42 to 60)	3,485 (3,300 to 3,687)	11 (7 to 16)
Maharashtra	5,243 (5,011 to 5,470)	78 (67 to 90)	3,390 (3,186 to 3,626)	14 (10 to 19)
UTs* other than Delhi	154 (147 to 161)	1 (1 to 1)	136 (128 to 145)	<1
**High ETL (152 million)**	**6,688 (6,406 to 6,964)**	**38 (34 to 42)**	**4,498 (4,221 to 4,804)**	**5 (3 to 8)**
Himachal Pradesh	385 (368 to 403)	7 (6 to 8)	212 (197 to 227)	1 (1 to 2)
Punjab	1,296 (1,241 to 1,351)	14 (12 to 18)	774 (721 to 829)	2 (2 to 4)
Tamil Nadu	3,206 (3,068 to 3,347)	30 (25 to 38)	2,004 (1,862 to 2,160)	6 (4 to 10)
Goa	67 (64 to 70)	<1	46 (43 to 48)	<1
Kerala	1,734 (1,658 to 1,805)	15 (12 to 19)	1,463 (1,381 to 1,550)	2 (2 to 4)

*UTs is Union territories. COPD is chronic obstructive pulmonary disease. ETL is epidemiological transition level.

4. Age-sex-specific prevalence of COPD and asthma in India, 2016

Age group (years)	COPD (95% uncertainty interval)		Asthma (95% uncertainty interval)	
	Men	Women	Men	Women
**1 to 4**	0.1 (0.1 to 0.1)	0.1 (0.1 to 0.1)	1.4 (1.2 to 1.7)	1.3 (1.1 to 1.6)
**5 to 9**	0.2 (0.2 to 0.2)	0.2 (0.2 to 0.2)	2.4 (2.0 to 2.9)	2.4 (2.0 to 2.9)
**10 to 14**	0.3 (0.3 to 0.3)	0.3 (0.3 to 0.3)	1.7 (1.5 to 2.0)	1.8 (1.5 to 2.1)
**15 to 19**	0.4 (0.4 to 0.5)	0.4 (0.4 to 0.4)	1.3 (1.1 to 1.6)	1.4 (1.2 to 1.7)
**20 to 24**	0.6 (0.5 to 0.6)	0.5 (0.5 to 0.5)	1.1 (0.9 to 1.3)	1.3 (1.0 to 1.5)
**25 to 29**	0.9 (0.9 to 1.0)	0.7 (0.7 to 0.8)	1.3 (1.1 to 1.4)	1.7 (1.5 to 1.9)
**30 to 34**	1.6 (1.5 to 1.7)	1.1 (1.0 to 1.2)	1.6 (1.4 to 1.9)	2.3 (1.9 to 2.7)
**35 to 39**	2.6 (2.4 to 2.8)	1.7 (1.5 to 1.8)	2.0 (1.7 to 2.2)	2.7 (2.4 to 3.1)
**40 to 44**	4.3 (4.0 to 4.7)	2.8 (2.6 to 3.0)	2.5 (2.1 to 2.9)	3.2 (2.7 to 3.8)
**45 to 49**	7.3 (6.7 to 7.9)	4.8 (4.4 to 5.2)	3.3 (2.9 to 3.7)	3.9 (3.4 to 4.5)
**50 to 54**	11.7 (10.8 to 12.7)	7.6 (7.0 to 8.3)	4.4 (3.8 to 5.1)	4.9 (4.1 to 5.7)
**55 to 59**	17.2 (16.0 to 18.5)	11.3 (10.5 to 12.1)	5.9 (5.1 to 6.7)	6.1 (5.3 to 7.0)
**60 to 64**	23.2 (21.7 to 24.7)	14.8 (13.8 to 15.8)	7.6 (6.5 to 8.9)	7.4 (6.2 to 8.7)
**65 to 69**	28.6 (26.9 to 30.3)	17.6 (16.5 to 18.7)	9.9 (8.8 to 11.2)	8.9 (7.8 to 10.3)
**70 to 74**	33.5 (31.6 to 35.5)	19.2 (18.0 to 20.4)	11.7 (10.1 to 13.5)	10.1 (8.5 to 11.8)
**75 to 79**	36.5 (34.5 to 38.6)	19.7 (18.5 to 21.0)	12.1 (10.7 to 13.5)	10.4 (9.1 to 11.9)
**80 plus**	37.8 (35.7 to 40.0)	18.9 (17.7 to 20.2)	11.7 (10.3 to 13.1)	10.1 (8.8 to 11.5)

COPD is chronic obstructive pulmonary disease.

5. Change in case-fatality rates of COPD and asthma in the states of India grouped by epidemiological transition level, 1990 to 2016

Disease conditions	State group	Crude case-fatality rate per hundred (95% uncertainty interval)			Age-standardised case-fatality rate per hundred (95% uncertainty interval)		
		1990	2016	Percent change 1990 to 2016 (95% uncertainty interval)	1990	2016	Percent change 1990 to 2016 (95% uncertainty interval)
**COPD**	Low ETL	2.46 (2.03 to 2.85)	1.86 (1.71 to 2.01)	-24.3 (-34.6 to -6.4)	3.57 (2.82 to 4.30)	2.49 (2.27 to 2.71)	-30.2 (-39.5 to -13.8)
	Lower-middle ETL	2.29 (1.91 to 2.59)	1.53 (1.41 to 1.62)	-33.3 (-42.6 to -19.6)	3.35 (2.70 to 3.96)	2.10 (1.95 to 2.22)	-37.3 (-45.6 to -24.5)
	Higher-middle ETL	2.09 (1.77 to 2.38)	1.30 (1.23 to 1.38)	-37.8 (-46.6 to -25.2)	3.11 (2.53 to 3.68)	1.77 (1.68 to 1.88)	-43.2 (-51.0 to -31.4)
	High ETL	1.69 (1.56 to 1.86)	0.99 (0.92 to 1.16)	-41.6 (-50.1 to -28.6)	2.43 (2.10 to 2.76)	1.25 (1.16 to 1.48)	-48.5 (-56.0 to -36.6)
	India	2.22 (1.88 to 2.53)	1.53 (1.44 to 1.63)	-30.9 (-39.2 to -17.2)	3.24 (2.65 to 3.83)	2.04 (1.91 to 2.17)	-37.0 (-44.5 to -24.2)
**Asthma**	Low ETL	1.06 (0.69 to 1.44)	0.62 (0.41 to 0.81)	-41.0 (-56.0 to -22.2)	1.70 (1.07 to 2.52)	0.88 (0.55 to 1.16)	-48.4 (-62.5 to -30.4)
	Lower-middle ETL	0.85 (0.58 to 1.11)	0.45 (0.32 to 0.55)	-46.7 (-61.1 to -30.7)	1.37 (0.89 to 1.88)	0.62 (0.41 to 0.75)	-54.9 (-68.0 to -40.1)
	Higher-middle ETL	0.80 (0.54 to 1.09)	0.38 (0.27 to 0.48)	-52.2 (-64.9 to -38.8)	1.30 (0.84 to 1.85)	0.50 (0.33 to 0.63)	-61.3 (-72.7 to -49.1)
	High ETL	0.62 (0.43 to 0.79)	0.27 (0.19 to 0.38)	-56.2 (-69.1 to -41.0)	0.90 (0.62 to 1.21)	0.31 (0.21 to 0.45)	-65.3 (-76.1 to -53.1)
	India	0.89 (0.61 to 1.22)	0.48 (0.33 to 0.62)	-45.9 (-59.0 to -31.0)	1.41 (0.52 to 2.06)	0.64 (0.43 to 0.83)	-54.8 (-66.7 to -41.2)

COPD is chronic obstructive pulmonary disease. ETL is epidemiological transition level.

6. Change in DALY rates of COPD and asthma in the states of India grouped by epidemiological transition level, 1990 to 2016

State group	COPD				Asthma			
	Crude rate 2016 (95% uncertainty interval)	Age-standardised rate 2016 (95% uncertainty interval)	Percent change in crude rate 1990 to 2016 (95% uncertainty interval)	Percent change in age-standardised rate 1990 to 2016 (95% uncertainty interval)	Crude rate 2016 (95% uncertainty interval)	Age-standardised rate 2016 (95% uncertainty interval)	Percent change in crude rate 1990 to 2016 (95% uncertainty interval)	Percent change in age-standardised rate 1990 to 2016 (95% uncertainty interval)
Low ETL	1,894 (1,728 to 2,096)	3,043 (2,761 to 3,360)	-10.9 (-21.5 to 6.1)	-30.3 (-38.4 to -17.3)	529 (380 to 697)	735 (515 to 975)	-44.2 (-55.3 to -30.9)	-49.9 (-61.3 to -36.6)
Lower-middle ETL	1,704 (1,562 to 1,872)	2,551 (2,342 to 2,767)	-9.8 (-20.5 to 4.8)	-35.6 (-42.9 to -25.2)	443 (337 to 546)	572 (429 to 704)	-45.0 (-56.5 to -32.1)	-54.2 (-65.0 to -42.4)
Higher-middle ETL	1,566 (1,453 to 1,688)	2,112 (1,954 to 2,281)	-13.8 (-23.9 to -1.2)	-42.3 (-48.8 to -33.6)	391 (302 to 496)	468 (358 to 595)	-47.3 (-58.6 to -36.6)	-59.1 (-68.9 to -49.5)
High ETL	1,292 (1,190 to 1,460)	1,443 (1,327 to 1,639)	-10.7 (-20.3 to 1.3)	-43.6 (-49.5 to -35.9)	299 (233 to 400)	312 (242 to 420)	-46.1 (-57.5 to -34.5)	-59.4 (-68.9 to -49.5)
India	1,700 (1,580 to 1,846)	2,432 (2,255 to 2,635)	-10.9 (-20.5 to 2.9)	-36.2 (-42.7 to -26.1)	450 (336 to 581)	566 (412 to 727)	-44.7 (-55.2 to -33.5)	-54.1 (-64.1 to -44.0)

DALY is disability-adjusted life-year. COPD is chronic obstructive pulmonary disease. ETL is epidemiological transition level.

7. Percent of COPD DALYs attributable to risk factors in the states of India grouped by epidemiological transition level, 2016

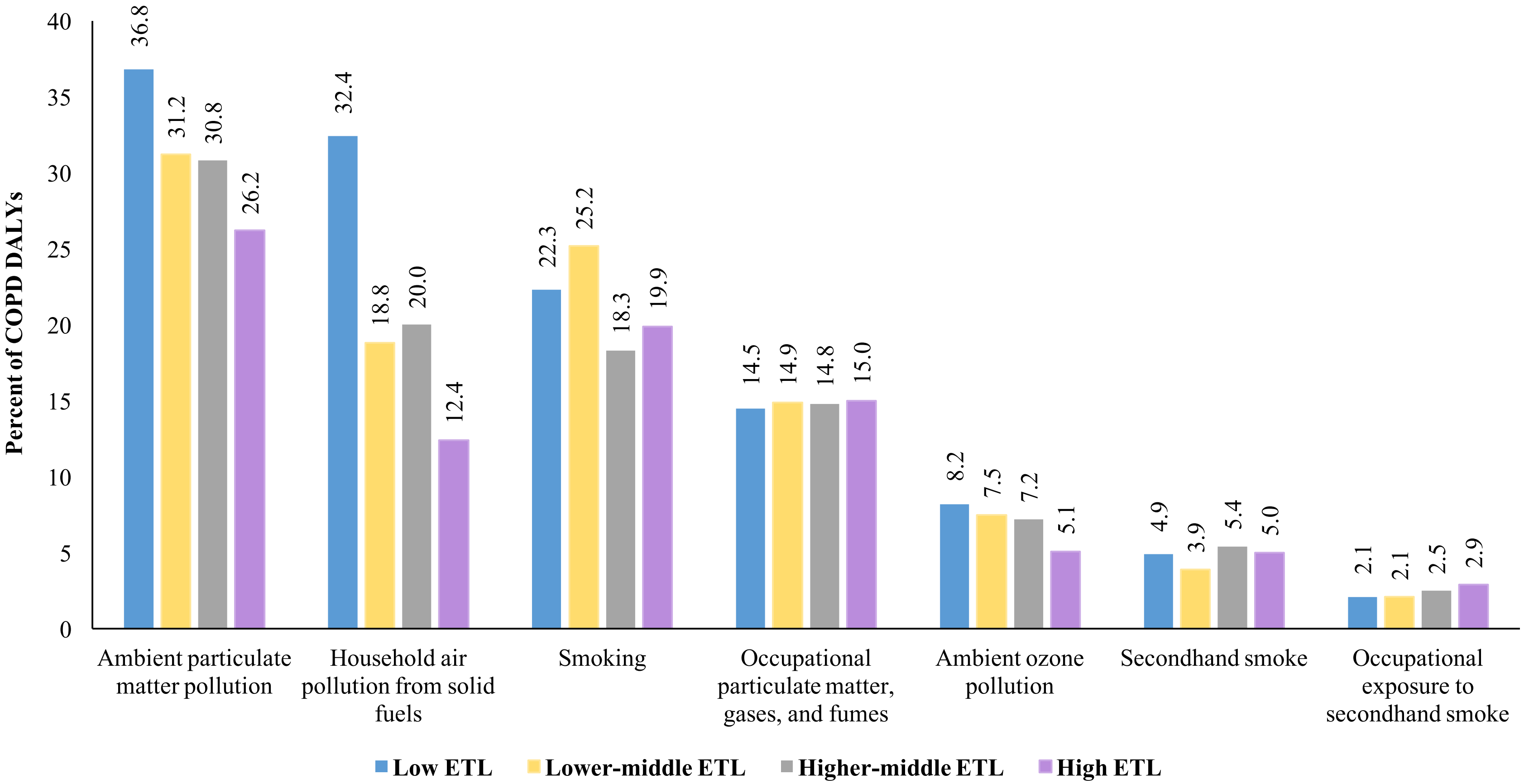

The cumulative impact of risk factors is not the simple addition of their individual contributions as the risk factors overlap, and also because the population attributable fractions from components can add up to more than their sum even if they are independent.
COPD is chronic obstructive pulmonary disease. DALY is disability-adjusted life-year. ETL is epidemiological transition level.

8. Percent of COPD DALYs attributable to risk factors in the states of India, 2016

States of India*	Ambient particulate matter pollution	Household air pollution from solid fuels	Smoking	Occupational particulate matter, gases, and fumes	Ambient ozone pollution	Secondhand smoke	Occupational exposure to secondhand smoke
Bihar	40.6	36.9	26.0	13.8	8.5	4.9	2.0
Jharkhand	34.5	31.9	9.6	13.3	8.0	7.2	3.8
Uttar Pradesh	40.1	32.6	24.0	14.7	9.0	4.3	1.8
Rajasthan	33.5	32.9	20.2	14.6	7.4	5.0	2.0
Meghalaya	27.3	21.6	33.4	14.0	4.1	3.9	1.4
Assam	29.2	27.2	26.7	14.5	5.2	4.0	1.9
Chhattisgarh	31.2	29.1	15.8	13.7	7.4	6.2	3.1
Madhya Pradesh	31.7	29.9	21.0	14.9	7.8	5.7	2.3
Odisha	31.6	29.0	14.4	13.3	6.9	6.0	2.7
Arunachal Pradesh	21.3	20.8	34.9	14.4	3.9	4.6	2.2
Mizoram	24.1	13.4	72.3	14.2	5.3	0.8	0.6
Nagaland	22.6	16.2	18.6	14.3	2.9	3.8	1.9
Uttarakhand	31.0	16.7	35.4	15.3	9.0	2.8	1.6
Gujarat	32.0	18.9	20.6	14.9	7.5	4.2	2.3
Tripura	29.4	25.8	31.6	14.6	5.7	5.1	2.0
Sikkim	24.6	13.7	34.2	15.2	5.4	4.4	2.5
Manipur	22.9	15.5	56.0	14.0	3.4	2.9	2.0
Haryana	39.0	19.0	26.9	15.7	7.2	4.6	1.9
Delhi	42.6	1.8	37.6	16.8	4.4	3.2	2.4
Telangana	27.9	24.3	7.7	14.0	6.5	6.5	2.8
Andhra Pradesh	26.7	21.7	10.0	14.3	6.1	5.5	2.3
Jammu and Kashmir	29.1	19.0	52.1	14.9	7.3	2.5	1.6
Karnataka	22.9	22.2	15.4	14.6	6.3	6.1	3.0
West Bengal	36.9	23.3	28.2	15.3	7.2	4.5	1.8
Maharashtra	30.1	17.0	10.7	14.7	8.5	6.0	2.9
UTs† other than Delhi	28.7	5.8	17.7	14.6	5.1	4.2	2.6
Himachal Pradesh	28.4	14.7	18.2	15.0	8.3	6.2	2.3
Punjab	37.2	13.1	17.8	15.6	6.9	6.8	4.1
Tamil Nadu	23.4	12.5	14.8	14.7	4.4	4.1	2.5
Goa	24.2	4.8	3.4	13.5	5.1	6.4	4.6
Kerala	21.4	11.0	33.8	15.0	4.1	4.9	2.6

*The states are listed in increasing order of epidemiological transition level in 2016. †UTs is union territories. COPD is chronic obstructive pulmonary disease. DALY is disability-adjusted life-year.

9. Comparison of age-standardised prevalence and DALYs per case of COPD and asthma in India versus the global average, 2016

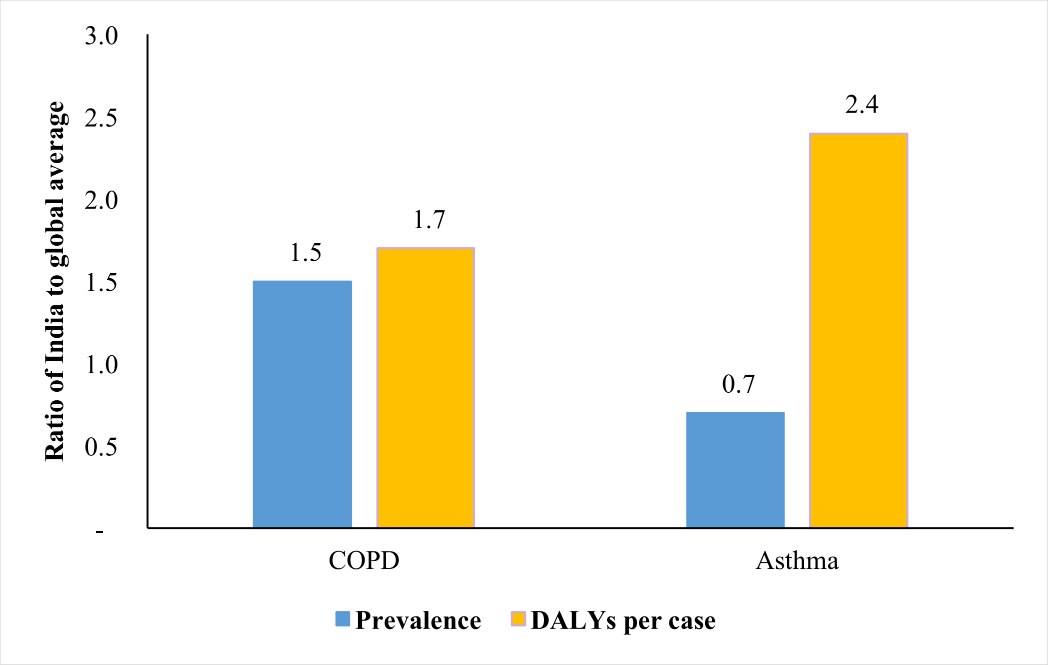

DALY is disability-adjusted life-year. COPD is chronic obstructive pulmonary disease.
